# Advances in the Use of Deep Learning for the Analysis of Magnetic Resonance Image in Neuro-Oncology

**DOI:** 10.3390/cancers16020300

**Published:** 2024-01-10

**Authors:** Carla Pitarch, Gulnur Ungan, Margarida Julià-Sapé, Alfredo Vellido

**Affiliations:** 1 Department of Computer Science, Universitat Politècnica de Catalunya (UPC BarcelonaTech) and Intelligent Data Science and Artificial Intelligence (IDEAI-UPC) Research Center, 08034 Barcelona, Spain; alfredo.vellido@upc.edu; 2 Eurecat, Digital Health Unit, Technology Centre of Catalonia, 08005 Barcelona, Spain; 3 Departament de Bioquímica i Biologia Molecular and Institut de Biotecnologia i Biomedicina (IBB), Universitat Autònoma de Barcelona (UAB), 08193 Barcelona, Spain; gulnur.ungan@autonoma.cat (G.U.); margarita.julia@uab.cat (M.J.-S.); 4 Centro de Investigación Biomédica en Red (CIBER), 28029 Madrid, Spain

**Keywords:** machine learning, neuro-oncology, radiology, deep learning, data analysis pipeline, ultra-low field magnetic resonance imaging

## Abstract

**Simple Summary:**

Within the rapidly evolving landscape of Machine Learning in the medical field, this paper focuses on the forefront advancements in neuro-oncological radiology. More specifically, it aims to provide the reader with an in-depth exploration of the latest advancements in employing Deep Learning methodologies for the classification of brain tumor radiological images. This review meticulously scrutinizes papers published from 2018 to 2023, unveiling ongoing topics of research while underscoring the main remaining challenges and potential avenues for future research identified by those studies. Beyond the review itself, the paper also underscores the importance of placing the image data modelling provided by Deep Learning techniques within the framework of analytical pipeline research. This means that data quality control and pre-processing should be correctly coupled with modelling itself, in a way that emphasizes the importance of responsible data utilization, as well as the critical need for transparency in data disclosure to ensure trustworthiness and reproducibility of findings.

**Abstract:**

Machine Learning is entering a phase of maturity, but its medical applications still lag behind in terms of practical use. The field of oncological radiology (and neuro-oncology in particular) is at the forefront of these developments, now boosted by the success of Deep-Learning methods for the analysis of medical images. This paper reviews in detail some of the most recent advances in the use of Deep Learning in this field, from the broader topic of the development of Machine-Learning-based analytical pipelines to specific instantiations of the use of Deep Learning in neuro-oncology; the latter including its use in the groundbreaking field of ultra-low field magnetic resonance imaging.

## 1. Introduction

Although Machine Learning (ML) is entering a phase of maturity, its applications in the medical domain at the point of care are still few and tentative at best. This paradoxical contradiction has been explained according to several different factors. One of them is the lack of experimental reproducibility, a requirement in which ML models in health have been reported to fare badly in comparison to other application areas [1]. One main reason to explain this is the mismatch between a data-centered (and often data-hungry) approach and the scarcity of publicly available and properly curated medical databases, combined with a nascent but insufficient data culture at the clinical level [2]. Another factor has to do with regulatory issues of ML (and Artificial Intelligence in general) in terms of both lack of maturity and geographical heterogeneity [3]. Further elements hampering ML-based tools adoption include data leakage, dataset shift, required model recalibrations, analytical pipeline maintenance failures, or changing medical practice patterns, to name a few [4].

The field of oncological radiology (and neuro-oncology in particular) is arguably at the forefront of the practical use of ML in medicine [5], now boosted by the success of Deep-Learning (DL) methods for the analysis of medical images [6,7]. Unfortunately, though, imaging does not escape the challenges and limitations summarized in the previous paragraph. Central to them, what has been called the “long-tail effect” [8]: pathologies for which only small and scattered datasets exist due to the scarcity of clinical data management strategies (technically complex and expensive) at levels beyond the local (regional, national, international). Associated with this, we must account for the difficulty of achieving standardized labeling (annotation) of imaging databases. An example of how to deal effectively with these problems is Federated Learning, which was used in [9] to gather data from 71 sites from 6 continents, analyzed using ML to address a problem of tumor boundary detection for glioblastoma brain tumors. Please note that the resulting database includes 6314 cases, which is impressive for this medical domain but still modest from an ML perspective. The success of ML in oncological radiology, as summarily stated in [10], will depend on its ability to create value in the delivery of medical care in terms of “increased diagnostic certainty, decreased time on task for radiologists, faster availability of results, and reduced costs of care with better outcomes for patients”.

This paper surveys some of the most recent advances in the use of ML for the analysis of magnetic resonance imaging (MRI) data in neuro-oncology without trying to make an all-encompassing review out of it. Instead, we focus on the most rapidly developing area, which involves the use of methods from the DL family. The variety of approaches sprouting from this family of methods has shaken the standards of data pre-processing or feature engineering before modeling as such. For this reason, we proceed to address the review hierarchically, starting in Section 3 with the broader topic of the development of ML-based analytical pipelines, which addresses the data analysis process beyond specific models and in which we will provide examples from two promising feature engineering approaches, namely source extraction in the form of independent component analysis (ICA) and nonnegative matrix factorization (NMF), and radiomics. The review of DL methods for image data analysis as such is delivered in Section 4. As an addition to this section, we will discuss the potential uses of DL in the groundbreaking field of ultra-low field (ULF) MRI [11]. Before all this, the following section will provide some contextual basic definitions of neuro-oncology concepts and a description of the main challenges and open issues concerning the use of ML in this domain.

## 2. Open Problems in AI Applied to MRI Analysis

The open problems for the use of ML-based analytical processes in the field of MRI in neuro-oncology can be seen from different perspectives. The first one is the analytical problem itself, according to which the main division is into categorization and segmentation problems. The latter is commented on later in this section.

Categorization can, in turn, be split into diagnosis and prognosis. In diagnosis, the correlation between neuroimaging classifications and histopathological diagnoses was assessed in [12] based on the 2000 version of the WHO classification of brain tumors and in [13] based on the 2007 version. In both studies, the main finding was that the sensitivity was variable among classes, whereas specificity was in the range of 0.85–1. The most difficult categories to diagnose were the glioma subtypes. The study based on the 2000 classification [12] reported a sensitivity of 0.14 for low-grade astrocytoma and 0.15 for low-grade oligodendroglioma. In the study based on the 2007 classification [13], increased sensitivity for low-grade astrocytoma (0.56) was found, but sensitivity was still low for other low-grade gliomas (LGG) such as oligodendroglioma (0.26), or for anaplastic gliomas (astrocytoma, 0.17 or ependymoma, 0.00), and other classes in the long-tail such as meningiomas of grade II and III in aggregate (0.17), or subependymomas and choroid plexus papilloma (0.33 for both). The recently released 2021 WHO classification [14], which incorporates the genetic alterations, opens the door to the reevaluation of these baseline results to accurately estimate the added value of any clinical decision support system (CDSS) based on ML or radiogenomics, over the limits of radiological interpretation of imaging findings. It is reasonable to foresee that the problematic tumor categories will remain so, or even more challenging, given the enhanced stratification of the glial category (e.g., different mutations of IDH1/2, ATRX, TP53, BRAF, H3F3A, CDKN2A/B, TERT and MGMT promoters, EGFR amplification, GFAP, 1p/19q codeletion, etc.).

On the other hand, regarding follow-up, there is no standard of care for recurrent high-grade gliomas, and the currently accepted criteria to assess response are those established by the Response Assessment in Neuro-Oncology Working Group (RANO) [15], which deals with the pseudoprogression phenomenon, defined as the appearance of contrast-enhancing lesions during the first 12 weeks after the end of the concomitant treatment or when the lesion developed within the first 3–6 months after radiation therapy, if it is in the radiation field (inside the 80% isodose line), and especially if it presents as a pattern of enhancement related to radiation-induced necrosis enhancement [16]. Also, with pseudoresponse in those patients treated with antiangiogenics in countries where these are approved [17,18]. Antiangiogenic agents, like bevacizumab, are designed to block the VEGF effect. The mechanism of action may be related to decreased blood supply to the tumor and normalization of tumor vessels, which display increased permeability. These agents are associated with high radiologic responses if we evaluate only the contrast enhancement. The recently published RANO 2.0 criteria [19] refine the former RANO, distinguishing between high-grade and low-grade gliomas. RANO 2.0 also takes into account the IDH status to decide whether the surrounding non-enhancing region should be taken into account or not. In this sense, ML-based pipelines should ideally be designed to allow the evaluation of their added value with respect to medical guidelines for clinical decision-making.

Another viewpoint to approach the open problems in the field has to do with the fact that ML-based analysis is strongly dependent on data pre-processing and the post-processing of results.

A fundamental prerequisite for the successful application of DL models in brain tumor classification is the pre-processing of the MRI data. The key pre-processing steps for the harmonization of MRI data are as follows.

- **Resampling**: MRI scans can exhibit variations in resolution and voxel sizes depending on the acquisition system. Resampling standardizes the resolution across the MRI images to ensure uniform dimensions.
- **Co-registration**: entails the alignment of MRI scans with a standardized anatomical template with the purpose of situating different scans within the same anatomical coordinate system.
- **Skull-stripping**: The main objective of the skull-stripping step is to efficiently isolate the cerebral region of interest from non-cerebral tissues, which enables DL models to focus exclusively on those brain tissues.
- **Bias Field Correction**: aims to rectify intensity inhomogeneities that are pervasive in MRI scans to guarantee uniformity in intensity values. The technique of choice for bias field correction is N4ITK (N4 Bias Field Correction) [20], which is an improved variant of the N3 (non-parametric nonuniform normalization) retrospective bias correction algorithm [21].
- **Normalization**: a technique adopted to rescale intensity values of MRI scans to a numeric range, rendering them consistent across the dataset. This process mitigates scale-related disparities. Two prominent approaches commonly applied to MRI data as input for DL models are min-max normalization and z-score normalization. Min-max achieves its goal by rescaling intensity values within MRI scans, spanning their range between 0 and 1. In contrast, z-score, often referred to as standardization, transforms the distribution of intensity values by centering it around a zero mean and standard deviation of value 1.
- **Tumor identification**: A critical and optional pre-processing step before the classification task involves identifying the tumor region of interest (ROI) through segmentation or by defining a bounding box that encompasses the tumor. Popular DL architectures, such as UNet [22], Faster-RCNN [23], and Mask-RCNN [24] are often employed to perform such segmentation or detection tasks.

The post-processing of results must often address the fact that the DL family of methods is, by their nature, an extreme case of black-box approach, a characteristic that may strongly hamper their medical applicability [25]. This limitation can be addressed using explainability and interpretability strategies; for further details on these, the reader is referred to [25].

## 3. Ml-Based Analytical Pipelines and Their Use in Neuro-Oncology

Ultimately, the whole point of using ML methods for data-based problems in the area of neuro-oncology is to provide radiologists with evidence-based medical tools at the point of care that can assist them in decision-making processes, especially with ambiguous or borderline cases. This is why it makes sense to embed these methods in Clinical Decision Support Systems (CDSS). A thorough and systematic review of intelligent systems-based CDSS for brain tumor analysis based on magnetic resonance data (spectra or images) is presented in this same Special Issue of Cancers [26]. It reports their increasing use over the last decade, addressing problems that include general ones such as tumor detection, type classification, and grading, but also more specific ones such as physicians’ alerting of treatment change plans.

At the core of ML-based CDSS, we need not just ML methods, models, and techniques but, more formally, ML pipelines. An ML pipeline goes beyond the use of a collection of methods to encompass all stages of the data mining process, including data pre-processing (data cleaning, data transformations potentially including feature selection and extraction, but also other aspects of data curation such as data extraction and standardization, missing data imputation and data clinical validation [27]) and models’ post-processing, potentially including evaluation, implementation and the definition of interpretability and explainability processes [25]. Pipelines can also accommodate specific needs, such as those related to the analysis of “big data”, with their corresponding challenges of standardization and scalability. As described in [28], in a clinical oncology setting, this may require a research infrastructure for federated ML based on the *findable*, *accessible*, *interoperable*, and *reusable* (FAIR) principles. Alternatively, we can aspire to automate the ML pipeline definition using Automated ML (AutoML) principles, as in [29], where Su and co-workers used a Tree-based Pipeline Optimization Tool (TPOT) in the process of selecting radiomics features predictive of mutations associated with midline gliomas.

An example of an ML pipeline for the specific problem of differentiation of glioblastomas from single brain metastases based on MR spectroscopy (MRS) data can be found in [30]. In this same issue of Cancers, Pitarch and co-workers [31] describe an ML pipeline for glioma grading from MRI data with a focus on the trustworthiness of the predictions generated by the ML models. This entails robustly quantifying the uncertainty of the models regarding their predictions, as well as implementing procedures to avoid data leakage, thus avoiding the risk of unreliable conclusions. All of these can be seen as part of a quest to avoid the pitfalls of implementation of ML-based CDSS that result in the problems of limited reproducibility of analytical results in clinical practice that have been reported in recent studies [1].

As previously explained, the first stages of an ML pipeline, prior to the data modeling itself, involve data pre-processing, and this task may, in turn, involve many sub-problems. As an example of the potential diversity and complexity of this landscape, we comment here on a few recently selected contributions to the problem of feature engineering and extraction following just two particular and completely different approaches: statistical image feature engineering using radiomics and source extraction using ICA- and NMF-based methods.

Radiomics is an image transformation approach that aims to extract either hard-coded statistical or textural features based on expert domain knowledge or feature representations learned from data, often using DL methods. The former may include first-order statistics, size and shape-based features, image intensity histogram descriptors, image textural information, etc. The use of this method for the pre-processing of brain tumor images prior to the use of ML has been recently and exhaustively reviewed in [32]. From that review, it is clear that the predominant problem under analysis is diagnosis, with only a limited number of studies addressing prognosis, survival, and progression. The types of brain tumors under investigation are dominated by the most frequent classes. In particular, glioblastoma, either on its own or combined with metastasis as a *super-class* of aggressive tumors, is the subject of many studies, with some others also including other frequent super-classes such as low-grade glioma or meningioma, while minority tumor types and grades are only considered in a limited number of studies. Importantly, and related to our previous comments concerning scarce data availability, most of the studies reported in [32] work with very small sample sizes, often not reaching the barrier of 100 cases. The challenge posed by data scarcity is compounded by the fact that most of the studies extract Radiomic features in the hundreds if not the thousands. This means that the ratio of cases-to-features is extremely low, making the use of conventional ML classifiers very difficult. To alleviate this problem, most of the reviewed papers resort to different strategies for qualitative and quantitative feature selection. Image modalities under analysis are dominated by T1, T2, and FLAIR, with few exceptions (PET, or Diffusion- and Perfusion-Weighted Imaging). Most studies are shown to resort to the Area Under the ROC Curve (AUC) as a performance metric, which is a safe choice, as it is far more robust than plain accuracy for small and class-imbalanced datasets.

The use of radiomics as a data transformation strategy in pre-processing is facilitated by the existence of off-the-shelf software such as the open-source PyRadiomics package [33].

Source extraction methods have a very different analytical rationale for data dimensionality reduction as a pre-processing step. They do not achieve it through plain feature transformation, as in radiomics. Instead, they aim to find the underlying and unobserved sources of observed radiological data. In doing so, they achieve dimensionality reduction as a byproduct of a process that may provide insight into the generation of the images themselves.

The ICA technique [34] has a long history in medical applications, most notoriously for the analysis of electroencephalographic signals. Source extraction is natural in this context as a tool for spatially locating sources of the EEG from electric potentials measured in the skull surface. In ICA, we assume that the observed data can be expressed as a linear combination of sources that are estimated to be statistically independent or as independent as possible. This technique has mostly been applied to brain tumor segmentation, but some alternative recent studies have extended its possibilities to dynamic settings, such as that in [35], where dynamic contrast-enhanced MRI is analyzed using temporal ICA (tICA), and in [36], where probabilistic ICA is used for the analysis of dynamic susceptibility contrast (DSC) perfusion MRI.

The NMF technique [37], on the other hand, was originally devised for the extraction of sources from images and assumes data non-negativity but does not assume statistical independence. Data are still approximated by linear combinations of factors. Although NMF and variants of this family of methods have extensively been used for the pre-processing and analysis of MRS and MRS imaging (MRSI) signal [38,39], they have only scarcely been used for the pre-processing of MRI. Some outstanding exceptions include the work in [40] with hierarchical NMF for multi-parametric MRI and the recent proposal of a whole new architecture based on NMF called Factorizer [41], constructed by replacing the self-attention layer of a Vision Transformer (ViT, [42]) block with NMF-based modules.

The technical details of ICA and NMF and their manifold variants are beyond the scope of this review and can be found elsewhere in the literature.

## 4. Deep Learning in Neuro-Oncology Data Analysis: A Review

In this section, we review existing recent literature to gather evidence about the advantages, challenges, and potential future directions in the use of DL techniques for supervised problems in neuro-oncology. Furthermore, we aim to provide insights into the current state-of-the-art methodologies, address their limitations, and identify areas for further research. Ultimately, our objective is to facilitate the development of robust, responsible, and applicable DL solutions that can effectively contribute to the field of neuro-oncology.

### 4.1. Overview of the Main DL Methods of Interest

Recent advances in the DL field have brought about new possibilities in medical imaging analysis and diagnosis. One of its arguably most successful models is Convolutional Neural Networks (CNNs), a widely used type of DL algorithm, well known for its ability to capture spatial correlations within image pixel data hierarchically. They have shown promise in medical imaging tasks [43,44,45], enabling improved tumor detection, classification, and prognosis assessment. The input data of a CNN is represented as a tensor with dimensions in the format of *(channels, depth, height, width)*. Notably, the “depth” dimension is specific to 3D images and not applicable to 2D data, and “height” and “width” correspond to the image’s spatial dimensions. In practical terms, the number of channels for color images is translated into three, representing Red, Green, and Blue (RGB) components, while gray-scale images consist of a single channel. The most characteristic operation in a CNN is called *convolution*, which gives the name to the convolutional layers. These layers capture spatial correlations by applying a set of filters or kernels across all areas of the input image data and compute the weighted sum, resulting in the generation of a feature map as an output. This feature map contains essential characteristics extracted by the actual layer and serves as the input for subsequent layers of processing. Another useful layer used in CNNs is the pooling layer. The pooling operation consists of downsampling the feature maps obtained from the convolution operation. The idea is to reduce the dimensionality without losing significant information. There are mainly two kinds of pooling: max-pooling and average-pooling. The outputs of convolutional layers are often passed through activation functions to introduce non-linearity. The most popular activation functions are ReLU, which inactivates negative values in the output through the formula f(x)=max(0,x); Sigmoid, which maps output values between 0 and 1 using the equation $f\left(x\right)=\frac{1}{1+e^{-x}}$; and SoftMax, which is the extension of Sigmoid for multi-class problems.

CNNs often consist of multiple layers that work together to learn hierarchical high-level image features. These layers progressively extract more abstract and complex information from the input image data. In the final step, the last feature map is passed through a fully connected layer, resulting in a one-dimensional vector. To obtain the class probabilities, Sigmoid or SoftMax are applied to this vector.

Several networks have made significant contributions to the world of DL. AlexNet [46], GoogLeNet [47], InceptionNet, VGGNet [48], ResNet [49], DenseNet [50], and EfficientNet [51] are among the most widely used CNNs to extract patterns from medical imaging.

DL models are considered data-hungry since they require substantial amounts of data for effective training. In the realm of medical data analysis, a primary challenge, as previously mentioned, is the inherent data scarcity and class imbalance. Common solutions to address this challenge include the application of data-augmentation (DA) methods and transfer-learning (TL) techniques.

Data Augmentation techniques are a crucial strategy to mitigate the challenge of limited annotated data in medical image analysis. These methods encompass a range of transformations applied to existing images, effectively expanding the dataset in terms of both size and diversity. Former approaches involve a wide range of geometric modifications such as rotation, scaling, flipping, cropping, zooming, or color changes. Beyond traditional augmentations, advanced methods like Generative Adversarial Networks (GANs) [52] are used to generate new synthetic and realistic examples.

The idea behind TL is to leverage pre-trained models, typically trained in large and diverse datasets, and adapt them for the specific task at hand, for which we might not have such a representative sample. Widely used pre-trained CNNs, such as ImageNet [53] or MS-COCO [54], have been originally developed from 2D large-scale datasets. However, a notable challenge when dealing with medical image data is the limited availability of large and diverse 3D datasets for universal pre-training [55]. Transferring the knowledge acquired from the 2D to the 3D domain proves to be a non-trivial task, primarily due to the fundamental differences in data structure and representation between these two contexts. To tackle this challenge and address the limitation of limited data, a broadly used strategy is to decompose 3D volumes into individual 2D slices within a determined anatomical plane. However, the decomposition of 3D volumes into individual 2D slices introduces a potential data leakage concern. This issue arises when 2D slices from the same individual inadvertently end up in both the training and testing datasets in an analytical pipeline. Such data leakage can lead to overestimations of model performance and affect the validity of experimental results. In addition, it is important to note that this approach comes with the trade-off of losing the 3D context present in the original data.

Recent efforts have aimed at overcoming these challenges. Banerjee et al. [56] classified low-grade glioma (LGG)and high-grade glioma (HGG) multi-sequence brain MRIs from TCGA and BraTS2017 data using multiple slice-based approaches. In their work, they provided a comparison of the performance obtained with CNNs trained from scratch on 2D image patches (PatchNet), entire 2D slices (SliceNet), and multi-planar slices through a final ensemble method that averages the classification obtained from each anatomical view (VolumeNet). The classification obtained with these models is also compared with pre-trained VGGNet and ResNet on ImageNet. The multi-planar method outperformed the rest of the approaches with an accuracy of 94.74%, and the lowest accuracy (68.07%) was obtained with pre-trained VGGNet. Unfortunately, TCGA and BraTS data share some patient data, which could involve an overlap between training and testing samples and hence be prone to data leakage. Ding et al. [57] combined radiomics and DL features using 2D pre-trained CNNs using single-plane images and performing a subsequent multi-planar fusion. VGG16, in combination with radiomics and RF, achieved the highest accuracy of 80% when combining the information obtained from the three views. Even though the multi-planar approach processes the information gathered from the axial, coronal, and sagittal views, it is still essentially a 2.5D approach, weak at fully capturing 3D contexts. Zhuge et al. [58] presented a properly native 3D CNN for tumor segmentation and subsequent binary glioma grade classification and compared it with a pre-trained 2D ResNet50 on ImageNet with previous tumor detection, employing a Mask R-CNN. The results of the 3D approach were slightly higher than the 2D ones, reporting 97.10% and 96.30% accuracy, respectively. In their study, Chatterjee et al. [59] explored the role of (2+1)D, mixed 2D–3D, and native 3D convolutions based on ResNet. This study highlights the effectiveness of mixed 2D–3D convolutions, achieving an accuracy of 96.98%, surpassing both the (2+1)D and the pure 3D approaches. Furthermore, the use of pre-trained networks demonstrated enhanced performance in the spatial models, yet, intriguingly, the pure 3D model performed better when trained from scratch. A study conducted by Yang et al. [55] introduced ACS convolutions, a novel approach that facilitates TL from models pre-trained on large publicly accessible 2D datasets. In this method, 2D convolutions are divided by channel into three parts and applied separately to the three anatomical views (axial, coronal, and sagittal). The effectiveness of this approach was demonstrated using a publicly available nodule dataset. Subsequently, Baheti et al. [60] further advanced the application of ACS convolutions by showcasing their enhanced performance on 3D MRI brain tumor data. Their study provides evidence of notable improvements in both segmentation and radiogenomic classification tasks.

### 4.2. Publicly Available Datasets

Access to large and high-quality datasets plays a crucial role in the development and evaluation of robust DL classification algorithms. This section aims to provide a comprehensive review of several publicly accessible datasets that have been widely used in brain tumor classification tasks and DL research. These datasets encompass diverse tumor types, imaging modalities, and annotated labels, facilitating the advancement of computational methods for accurate tumor classification.

Table 1 provides a detailed overview of the most frequently used datasets in the literature.

The Brain Tumor Segmentation Challenge (BraTS) and The Computational Precision Medicine: Radiology-Pathology Challenge on Brain Tumor Classification (CPM-RadPath) datasets were created for two popular challenges held at the MICCAI (Medical Image Computing and Computer Assisted Intervention) Conference.

The BraTS Challenge [61] was initially developed in 2012 to benchmark tumor segmentation methods distinguishing glioblastoma from “lower grades”. Notably, this challenge provides not only MRI data but also clinical labels, including a binary classification of glioma grades. Even though their definition does not fully align with WHO’s terminology, they include grades 2 and 3 when referring to “lower grades”.

Throughout the years, the BraTS Challenge has continually evolved, expanding to include additional tasks and diverse datasets. In 2017, the dataset was enriched by integrating data from the TCIA repository, specifically including samples from the TCGA-LGG [71] and TCGA-GBM [70] datasets. It is worth noting that TCGA-LGG data provides labels to differentiate between gliomas of grades 2 and 3. Although the primary focus of the BraTS Challenge has traditionally centered on automated brain tumor segmentation, it has grown to become a widely adopted resource for brain tumor grade classification. Recent challenges have included tasks such as survival prediction and genetic classification, and the 2023 challenge even included image synthesis tasks.

CPM-RadPath [62] from 2019 was designed to evaluate brain tumor classification algorithms in three classes, taking into account the WHO classification of 2016: A (astrocytomas grades II and III, IDH-mutant), O (oligodendroglioma grades II and III, IDH-mutant, 1p/19q codeleted) and G (Glioblastoma and diffuse astrocytic glioma with molecular features of glioblastoma, IDH-wildtype (Grade IV)), interestingly grouping the anaplasic with the low grades in the A and O classes.

This challenge provides participants with paired radiology scans and digitized histopathology images. It is worth noting that the data provided by these challenges are distributed *after* pre-processing, involving co-registered to the same anatomical template, interpolated to a consistent resolution of 1 mm^3^, and skull-stripped.

The datasets under consideration encompass a variety of MRI modalities. Specifically, BraTS, CPM-RadPath, REMBRANDT, and TCGA comprise images from four key modalities: T1, T1 post-contrast weighted (T1c), T2-weighted, and Fluid Attenuated Inversion Recovery (FLAIR). The IXI dataset provides not only T1 and T2 but also Proton Density (PD) and Diffusion-weighted (DW) images. Notably, images on Figshare are limited to the T1c modality, while datasets from Kaggle and Radiopaedia lack this information.

The images in the BraTS, CPM-RadPath, IXI, REMBRANDT, and TCGA datasets are stored in 3D structures using widely used medical image formats, specifically NIfTI or DICOM. In contrast, datasets sourced from Kaggle consist of 2D images in PNG format. Notably, Figshare contains 2D images in MATLAB data format. In the Figshare data repository, images are provided alongside a 5-fold CV split at the patient level to mitigate the risk of data leakage. The use of this split ensures that no patient is inadvertently present in both training and testing sets, thus preventing leakage. Moreover, this dataset comprises multiple 2D slices from the same patient in the three distinct anatomical perspectives. Conversely, the datasets sourced from the Kaggle repository lack patient identifier information, making it challenging to ascertain if images are from unique patients or to trace the origin of the data.

**Figure 1:**
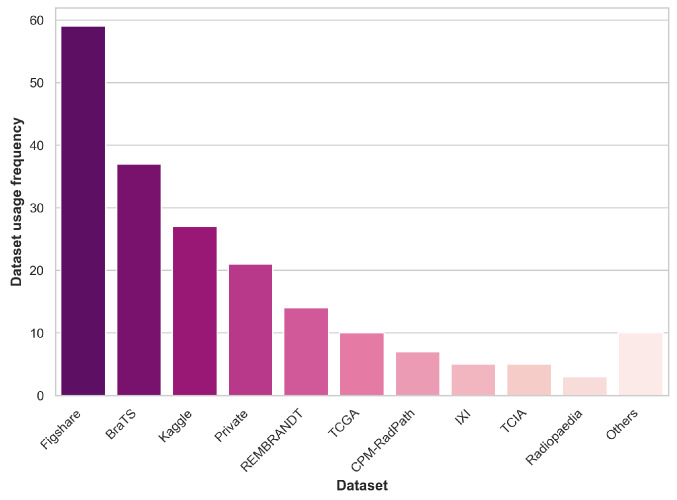
Dataset usage prevalence across the reviewed literature.

Figure 1 summarizes the prevalence of dataset usage in the reviewed literature, including public and private datasets. Datasets that appear in two or fewer papers are grouped under the “Others” category.

It is worth highlighting that over 85% of the papers reviewed in this analysis make use of public datasets. It is essential to acknowledge that the sample sizes of the datasets, in general, are roughly in the hundreds range. This limited sample size can pose challenges in drawing robust and generalizable conclusions, which is a notable concern within the ML healthcare domain. Addressing the need for larger and more diverse datasets, as previously discussed, is an ongoing challenge in this field.

### 4.3. Literature Review

**Figure 2:**
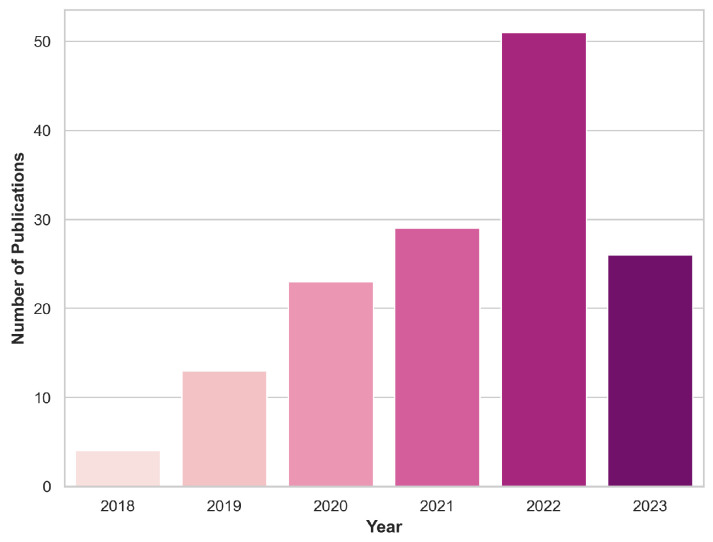
Yearly inclusion of articles in this review that focus on classifying brain tumors using DL and MRI scans.

Various online repositories of scientific research articles, including PubMed, Google Scholar, and Scopus, were utilized to collect pertinent papers for this review section. The selection was restricted to the years 2018–2023. More specifically, only articles published prior to 30 June 2023 were taken into consideration. The document type was restricted to journal or conference papers. The focal keywords were centered on classifying brain tumors from pre-operative MRI images using DL techniques. While refining our choices, we excluded publications with ambiguous data explanations or lacking methodology details, as the utmost priority was placed on guaranteeing the strength and acuity of our conclusions. An initial identification process yielded a total of 555 papers, with 146 papers remaining after the screening procedure. Figure 2 depicts the distribution of these papers across the years under review, shedding some light on the temporal evolution of research in this domain.

In the subsequent analysis, we provide comprehensive insights into the data sources and methodologies employed in the examined papers. Table A1 offers a detailed overview of the datasets, focusing on essential aspects such as the dimensionality of the images, sample size, MRI details, and pre-processing methods used. Table A2 delves into the specifications of the employed DL models, highlighting the brain tumor classification task, data partitioning, architecture, and the reported performance metrics. These tables contribute to a comprehensive understanding of the methodologies employed in the reviewed literature. Table A1 and Table A2 exclusively display the information available from the original authors in the analyzed papers. Any omissions in the table reflect the absence of such details as provided by the original authors in the surveyed papers. Notice that several papers are marked with an asterisk (*), which denotes that not all models have been reported in our tables due to the extensive array of results reported by the authors. Especially in these cases, we recommend readers refer to the original papers for a comprehensive overview of findings.

In this review, we focus on the differentiation of primary brain tumor types, with particular attention to gliomas due to their aggressive nature. Among the 146 examined papers in this section, some address multiple tasks concurrently. Specifically, 77 focused on distinguishing primary brain tumor types, while 27 aimed to identify tumorous images from images of healthy patients. Furthermore, the pursuit of accurate glioma grade classification is assessed in 66 papers, with 41 of them focusing on the binary distinction between low (grades II and III) and high-grade (glioblastoma, grade IV) gliomas. Note that the question asked by these 41 works does not correspond to any of the canonical releases of the WHO classification of brain tumors, as III is, in fact, high-grade. In this sense, such a grouping would facilitate the achievement of good performance results by grouping entities that are more prone not to show contrast enhancement, in contrast to glioblastoma, which always will show contrast enhancement [72]. Additionally, 12 studies delve into the distinction of glioma subtypes.

As previously highlighted, pre-processing techniques are pivotal in medical image processing. Among the 146 papers analyzed in this review, a substantial 80% of them provide insights into the specific pre-processing methodologies that were employed. Within this subset, it was observed that 35% employed registration techniques involving registration to a common anatomical template and co-registration to the same MRI modality. Furthermore, 40% employed segmentation as a critical step to isolate the brain from the surrounding skull structures. Notably, nearly half of the papers embraced normalization techniques to standardize the intensity of the image data before it was fed to the models. Additionally, 30% of the papers undertook the task of brain tumor extraction through methods such as bounding box delineation or tumor segmentation. Moreover, 15% of the papers employed pre-processing integrated image enhancement techniques to improve the contrast and visibility of crucial anatomical structures. In several studies [73,74,75], researchers investigated the advantages of utilizing the tumor area as opposed to the entire image, highlighting the significant benefits of concentrating on the tumor region rather than the entire image.

In the realm of medical research, the size and diversity of the training data sample stand as fundamental factors that substantially influence the performance, generalizability, and robustness of ML models. Several studies have explored the impact of varying the size of the training data sample on model performance [76,77,78,79,80,81,82,83,84]. Their findings highlight the value of ensuring that a substantial volume of data is available for training, as it significantly contributes to the model’s ability to make more accurate and reliable predictions.

Regarding addressing the limitation of data scarcity, approximately 60% of the examined studies employed DA techniques, and 40% incorporated TL in a 2D domain as a viable solution. Several of these investigations [85,86,87,88,89,90,91,92] have demonstrated the advantages of increasing both the quantity and variability of the samples through the inclusion of augmented images. Applying traditional DA techniques, such as geometric variations from original images, was the most widely used strategy, while only a few studies opted for the use of DL generative models [89,93,94,95]. Several studies [73,92,96,97,98,99] have integrated DA as an oversampling technique to address the problem of imbalanced data in the context of brain tumor classification. Furthermore, other works have explored the inclusion of multi-view 2D slices from axial, coronal, and sagittal planes, in addition to employing image flipping and rotations to augment the dataset [100]. Pre-trained models have demonstrated performance enhancements in the classification of glioma grades in several studies [79,101,102]. However, it is noteworthy that not all investigations have reported equivalent advantages when employing pre-trained models to discriminate between healthy and tumorous samples [103] or to differentiate tumor types [104]. These variations in findings underscore the complexity of the observed performance disparities, which may not be solely ascribed to the classification task itself but may also be influenced by intrinsic dataset variations.

The ability of CNNs to automatically extract meaningful features from brain MRI images, as opposed to the conventional need for manual feature engineering in certain ML algorithms like RF, GrB, and SVM, has been emphasized by numerous studies. These studies underscore the potential of CNNs in revolutionizing the landscape of MRI feature extraction for enhanced accuracy and efficiency in brain tumor classification [105,106,107,108,109,110,111]. Most of the reviewed papers (approximately 60%) utilized established state-of-the-art CNN architectures to obtain brain tumor classification. Among these, ResNet and VGGNet backbones were the most prevalent choices, closely followed by AlexNet, GoogLeNet, and Inception. In contrast, the remaining 40% of the papers concentrated on enhancing brain tumor classification by introducing novel model architectures. The inherent black-box nature of CNNs highlights the importance of delving into the comprehension of their predictions, especially in a medical context. Several studies within our review [74,112,113,114,115,116,117] have applied post-processing explainability tools to validate that the network’s decision-making process aligns with the intended diagnostic criteria, therefore enhancing the reliability of CNN-based medical applications.

Additionally, selected studies [57,118,119] explored the synergies of ensemble learning by combining the outputs of radiomics and DL models. Another interesting area of research has considered the opportunity of incorporating ML classifiers as the final layer in CNNs, effectively bypassing the traditional SoftMax layer [76,96,99,103,119,120,121,122,123,124,125,126,127,128].

The integration of information from various data sources has garnered growing interest in the medical field. Brain tumors, due to their distinct features both at the histopathological and radiological level, have motivated numerous studies to explore the synergy between whole slide imaging (WSI) and MRI data [97,129,130,131,132]. These investigations consistently highlight the richer information content in WSI as compared to MRI. However, they also reveal that combining data from both sources leads to improved overall performance in brain tumor characterization. Ensemble learning methods have shown promise in not only integrating information from diverse data types but also in combining predictions from multiple DL models on MRI to improve overall performance [75,82,91,107,108,116,122,133,134,135,136,137,138,139]. As brain tumor diagnosis and prognosis are significantly linked to genetic factors, several studies have undertaken efforts to explore the capabilities of DL models in extracting meaningful MRI features for the classification of these genetic frameworks [56,83,93,100,117,140,141].

Although brain MRIs inherently capture 3D data, a notable observation is that over 80% of the studies conducted their analyses within a 2D domain, focusing on 2D MRI slices. Nonetheless, some investigations have actively explored the significance of incorporating 3D volumetric information into the realm of brain tumor classification [56,58,59,74,97,98,100,112,117,129,130,131,132,135,140,141,142,143,144,145,146,147,148]. Although 3D volumes inherently capture information from the three anatomical planes, 2D slices are restricted to a specific view. Notably, among the studies that adopted a 2D approach, only 44% provided details about the chosen anatomical plane. Among this subset, more than 50% utilized axial, coronal, and sagittal views, while over 40% exclusively employed axial views.

Similarly, close to 70% of the reviewed studies disclosed the MRI modalities utilized for the analysis. Among these, close to 50% exclusively employed the T1c sequence, while 26% used a combination of T1c, T1, T2, and FLAIR sequences, 12% used three sequences, and the rest chose one sequence. Various strategies were employed to integrate information from multiple modalities. The prevalent method involved fusing them as input channels, comparable to the treatment of channels in RGB images. In their study, Ge et al. [100] evaluated the sensitivity of T1c, T2, and FLAIR modalities in glioma grade classification. Their investigation highlighted the T1c sequence as the most informative among these modalities. To further enhance the classification performance, they incorporated information from each source using an aggregation layer within the network architecture. Subsequently, similar ensemble learning approaches were adopted by Gutta et al. [106], Hussain et al. [148], Rui et al. [149]. Notably, Guo et al. [150] directly compared the performance of a modality-fusion approach, where the four MRI modalities were concatenated as a four-channel input, with a decision-fusion approach, where final predictions were derived through a linear weighted sum from the probabilities obtained through four independent pre-trained unimodal models. This study reinforced the notion of the T1c modality’s significance in glioma subtype classification. Moreover, it revealed that any multimodal approach consistently outperformed unimodal models, with the decision-ensemble approach emerging as the most effective strategy.

As previously discussed, decomposing 3D volumes into individual 2D slices may introduce the potential for data leakage. Maintaining the reliability of the analysis is crucial for obtaining robust and trustworthy findings. It is worth noting, however, that only a limited number of studies that use multiple 2D slices [56,76,77,79,93,100,101,106,107,115,118,126,135,149,151,152,153,154,155,156,157], explicitly detailed their approach to data splitting at the patient level, addressing this critical concern. Remarkably, an insightful comparison was carried out in the work of Badža and Barjaktarović [158] between data-splitting strategies at the patient and image levels. The findings elucidate that an image-wise approach yields accuracy results as high as 96% for brain tumor type classification, while a patient-level split demonstrates a higher degree of reliability with an accuracy of 88%. These results underscore the critical importance of utilizing a patient-wise training approach to assess the model’s generalization capacity. Similarly, Ghassemi et al. [85], Ismael et al. [159] also provided evidence of superior performance when using an image-wise split, further reinforcing the importance of thoughtful data splitting. It is also important to note that 3D models operate on complete 3D volumes and are inherently structured at the patient level. This approach substantially reduces the likelihood of data leakage, therefore enhancing the reliability of the analysis and ensuring that the results faithfully represent the model’s performance. This aspect may provide a valuable perspective when interpreting differences in accuracy between 3D and 2D models.

The predominant approach for data partitioning in the examined papers involves the use of hold-out validation with training and validation sets. This was followed by the adoption of K-fold cross-validation, which enhances the robustness of model evaluation. A less frequently employed method was the three-way split, which includes training, validation, and testing sets. In total, only 36% of the studies assessed their final results using an independent test set. Decuyper et al. [140], Gilanie et al. [152], Alanazi et al. [160] took a step further by assessing the generalizability and robustness of their models using external validation sets. Although the authors of the Figshare dataset thoughtfully included a 5-fold CV setup alongside the data to promote comparability and reproducibility, it is still important to remark here that a substantial majority of studies continue to prefer custom data partitioning methods.

## 5. Machine Learning Applications to Ultra-Low Field Imaging

A completely different area of application of ML to neuroradiology has recently emerged with the availability of ultra-low field magnetic resonance imaging devices for point-of-care applications, typically with <0.1 T permanent magnets [161,162,163]. In the 0.055 T implementation described by Liu et al. [11], DL was used to improve the quality of the acquisition by detecting and canceling external electromagnetic interference (EMI) signals, eliminating the need for radio-frequency shielded rooms. They compared the results of the DL EMI cancelation in 13 patients with brain tumors, both in the 0.055 T and in another 3 T machine, on same-day acquisitions, finding that it was possible to identify the different tumor types. Please note that these processes are, in fact, a completely different use of DL for data pre-processing to those reviewed in Section 3.

Another example is the *Hyperfine* system, which received FDA clearance in 2020 for brain imaging and in 2021 (K212456) for DL-image reconstruction to enhance the quality of the generated images. In particular, DL is used as part of the image reconstruction pipeline of T1, T2, and FLAIR images. There are two DL steps: the first one is a so-called DL gridding, where the undersampled k-space data are transformed into images not by Fourier transformations but with DL. The transformed images are then combined, and a final post-processing DL step is applied to eliminate noise. However, no details about the specific algorithms are provided. Although the main application seems to be in the neurocritical setting [164], this system is beginning to be compared with the imaging quality at higher fields at different stages, with a particular interest in the early post-operative monitoring after surgical resection (e.g., [165,166]). It is to be expected that *Hyperfine* brain tumor applications will emerge soon, for example, through the partnership with The Brain Tumor Foundation, to provide the general population with free brain scans.

## 6. Conclusions

Neuro-oncological radiology relies on non-invasive data acquisition, which makes it the ideal target of data-centered analytics and places it at the forefront of ML-applied developments. In this review paper, we have focused on the most successful instantiation of ML currently, namely DL, and its use for the analysis of imaging data. Emphasis has been put on the fact that DL methods must be seen as only part of analytical pipelines, in which data pre-processing plays a key role.

Promoting the responsible utilization of clinical data is of utmost importance when striving to establish trustworthy conclusions. A fundamental step in this endeavor is the comprehensive disclosure of both the data used and the analytical procedures undertaken. Such transparency not only fosters greater trust in research outcomes but also amplifies the generalizability and reproducibility of the findings. This, in turn, plays a pivotal role in advancing AI-driven solutions in the clinical pathway. Most DL-based analytical solutions depend, to a great extent, not only on data quality but also quantity. For this reason, we argue that the main challenge facing the use of DL in the radiological imaging setting is precisely the creation of sizeable curated image databases for the different problems at hand.

## Figures and Tables

**Table 1:** An overview of publicly available MRI datasets for brain tumor classification benchmarking.

Dataset		Categories	Dim.	Sample Size	MRI Modalities
BraTS [61]	2020	Low-Grade Glioma (LGG) High-Grade Glioma (HGG)	3D	369 (LGG: 76, HGG: 293)	T1, T1c, T2, FLAIR
	2019		3D	335 (LGG: 76, HGG: 259)	T1, T1c, T2, FLAIR
	2018		3D	284 (LGG: 75, HGG: 209)	T1, T1c, T2, FLAIR
	2017		3D	285 (LGG: 75, HGG: 210)	T1, T1c, T2, FLAIR
	2015		3D	274 (LGG: 54, HGG: 220)	T1, T1c, T2, FLAIR
	2013		3D	30 (LGG: 10, HGG: 20)	T1, T1c, T2, FLAIR
	2012		3D	30 (LGG: 10, HGG: 20)	T1, T1c, T2, FLAIR
CPM-RadPath [62]		Astrocytoma (AS) IDH-mutant Oligodendroglioma (OG) IDH-mutant 1p/19q codeletion Glioblastoma (GB) IDH-wildtype	3D	Training: 221 (AS: 54, OG: 34, GB: 133) [unseen sets] Val: 35, Test: 73	T1, T1c, T2, FLAIR
Figshare [63]		Meningioma (MN), Glioma (GL), Pituitary (PT)	2D	233 (MN: 82, GL: 89, PT: 62)	T1c
IXI [64]		Healthy	3D	600	T1, T2, PD, DW
Kaggle-I [65]		Healthy (H), Tumor (T)	2D	3000 (H: 1500, T: 1500)	-
Kaggle-II [66]		Healthy (H), Meningioma (MN), Glioma (GL), Pituitary (PT)	2D	3264 (H: 500, MN: 937, GL: 926, PT: 901)	-
Kaggle-III [67]		Healthy (H), Tumor (T)	2D	253 (H: 98, T: 155)	-
Radiopaedia [68]		-	-	-	-
REMBRANDT [69]		Oligodendroglioma (OG), Astroctyoma (AS), Glioblastoma (GB)	3D	111 (OG: 21, AS: 47, GB: 44)	T1, T1c, T2, FLAIR
		Grade II (G.II), Grade III (G.III), Grade IV (G.IV)		109 (G.II: 44, G.III:24, G.IV: 44)	
TCGA-GBM [70]		Glioblastoma	3D	262	T1, T1c, T2, FLAIR
TCGA-LGG [71]		Grade II (G.II), Grade III (G.III)	3D	197 (G.II: 100, G.III: 96, discrepancy: 1)	T1, T1c, T2, FLAIR
		Astroctyoma (AS), Oligodendroglioma (OG), Oligoastrocytoma (OAS)		197 (AS: 64, OG: 86, OAS: 47)	

DW: Diffusion-weighted, FLAIR: Fluid Attenuated Inversion Recovery, PD: Proton Density, T1c: contrast-enhanced T1 weighted.

## Data Availability

Data sharing is not applicable.

## Abbreviations

The following abbreviations are used in this manuscript:

AUC	Area Under the ROC Curve
CDSS	Clinical Decision Support System
CNN	Convolutional Neural Network
DA	Data Augmentation
DL	Deep Learning
FAIR	Findable, Accessible, Interoperable, Reusable
GAN	Generative Adversarial Network
HGG	High-Grade Glioma
ICA	Independent Component Analysis
LGG	Low-Grade Glioma
ML	Machine Learning
MRI	Magnetic Resonance Imaging
MRS	Magnetic Resonance Spectroscopy
MRSI	Magnetic Resonance Spectroscopy Imaging
NMF	Nonnegative Matrix Factorization
RF	Random Forest
RGB	Red, Green, Blue
ROI	Region of Interest
SVM	Support Vector Machine
TCGA	The Cancer Genome Atlas
TCIA	The Cancer Imaging Archive
tICA	Temporal ICA
TL	Transfer Learning
TPOT	Tree-based Pipeline Optimization Tool
ULF	Ultra-Low Field
ViT	Vision Transformer
WHO	World Health Organization
WSI	Whole Slide Imaging

## Appendix A. Literature Review Summary of Deep-Learning Sources

This appendix comprises Table A1 and Table A2, offering a detailed overview of the datasets and models employed in the papers summarized in Section 4.3. Table A1 provides key details about the datasets used, including image dimensionality, number of patients in the study and images included for the analysis, anatomical plane, MRI modalities, pre-processing procedures, and data-augmentation techniques. On the other hand, Table A2 summarizes analytical aspects, such as the classification task, data-splitting methodology, network architecture, overall performance metrics, and class-specific performance. Hyphens (-) in certain cells indicate that the information was not provided in the original paper.

For papers marked with an asterisk (*), not all outcomes are included in the table due to the extensive range of results reported by the authors. We encourage readers to consult the original papers for a more comprehensive understanding of the findings.

**Table A1:** Data Overview: Comprehensive overview of the datasets examined within the DL literature review centered on brain tumor classification tasks and MRI data. Essential information regarding dimensionality, sample size, anatomical plane, MRI modalities, and pre-processing methods are summarized.

No.	Reference	Dim.	Dataset	Sample Size		Plane	MRI Modality	Pre-Processing	Data Augmentation (Augmentation Factor)
				Patients	Images				
1	Ge et al. [100] (2018)	2D	BraTS2017	285 (Table 1)	-	Ax, Sag, Cor	T1c, T2, Flair	Tumor mask enhancement	Multi-view images (ax, sag, cor), rotation, flipping
2	Ge et al. [73] (2018)	3D	BraTS2017	285 (Table 1)	285 (Table 1)	Ax, Sag, Cor	T1c	None ^1^ Tumor mask enhancement ^2^	(LGG: 2) Flipping
3	Pereira et al. [74] (2018)	3D	BraTS2017	285 (Table 1)	285 (Table 1)	Ax, Sag, Cor	T1, T1c, T2, Flair	BFC, z-score normalization (inside brain mask)	Sagittal flipping, rotation, exponential intensity transformation
4	Yang et al. [101] (2018)	2D	Private	113 (LGG: 52, HGG: 61)	867 (LGG: 368, HGG: 499)	Ax	T1c	Z-score normalization, tumor ROI	(14) HE, random rotation, zooming, adding noise, flipping
5	Abd-Ellah et al. [167] (2019)	2D	Brats2017	-	1800 (H: 600, LGG: 600, HGG:600)	-	-	-	-
6	Anaraki et al. [168] (2019)	2D	IXI REMBRANDT TCGA-GBM TCGA-LGG Private Figshare	600 130 199 299 60 233	16,000 (H: 8000, G.II: 2000, G.III: 2000, G.IV: 4000) 989	Ax - - - - Ax	T1 T1c T1c T1c T1c T1c	Normalization, resizing	Rotation, translating, scaling, flipping
7	Deepak and Ameer [76] (2019)	2D	Figshare	233 (Table 1)	3064 (Table 1)	Ax, Sag, Cor	T1c	Min-max normalization, resizing	Rotation, flipping
8	Hemanth et al. [169] (2019)	2D	Private	-	220	-	T1, T2, Flair	None	None
9	Kutlu and Avcı [120] (2019)	2D	Figshare	233 (Table 1)	3064	Ax, Sag, Cor	T1c	None	None
10	Lo et al. [102] (2019)	2D	TCIA	134 (G.II: 30, G.III: 43, G.IV: 57)	134 (G.II: 30, G.III: 43, G.IV: 57)	Ax	T1c	Normalization, CE, tumor segmentation	(56) AutoAugment [170]
11	Muneer et al. [171] (2019)	2D	Private	20	557 (G.I: 130, G.II: 169, G.III: 103, G.IV: 155)	Ax	T2	Skull-stripping, tumor segmentation	Resize, reflection, rotation
12	Rajini [172] (2019)	2D	IXI REMBRANDT TCGA-GBM TCGA-LGG Figshare	600 130 “around 200” 299 233	- - - - -	- - - - -	- - - - T1c	- - - - -	- - - - -
13	Rahmathunneesa and Muneer [173] (2019)	2D	Private	-	760 (G.I: 198, G.II: 205, G.III: 172, G.IV: 185)	Axial	T2	Skull-stripping, resizing	Resizing, rotation, translation, reflection
14	Sajjad et al. [174] (2019)	2D	Radiopaedia Figshare	- 233	121 (G.I: 36, G.II: 32, G.III: 25, G.IV: 28) 3064 (Table 1)	- -	- T1c	BFC, Segmentation, Z-score normalization	(30) Rotation, flipping, skewness, shears, gaussian blur, sharpening, edge detection, emboss
15	Sultan et al. [175] (2019)	2D	Figshare REMBRANDT	233 (Table 1) 73 (G.II: 33, G.III: 19, G.IV: 21)	3064 (Table 1) 516 (G.II: 205, G.III: 129, G.IV: 182)	Ax, Sag, Cor	T1c	Resizing	(5) Rotation, flipping, mirroring, noise
16	Swati et al. [77] (2019)	2D	Figshare	233 (Table 1)	3064 (Table 1)	Ax, Sag, Cor	T1c	Min-max normalization, resizing	-
17	Toğaçar et al. [176] (2019)	2D	Kaggle-III	-	253 (Table 1)	-	-	-	Rotation, flipping, brightening, CE, shifting, scaling
18	Amin et al. [177] (2020)	2D	BraTS2012 ^1^ BraTS2013 ^2^ BraTS2013 (LB) ^3^ BraTS2015 ^4^ BraTS2018 ^5^	25 (LGG: 5, HGG: 10) 30 (Table 1) 25 (LGG: 4, HGG: 21) 274 (Table 1) 284 (Table 1)	- - - - -	-	T1, T1c, T2, Flair	Noise Removal, tumor enhancement, MRI modality fusion	-
19	Afshar et al. [178] (2020)	2D	Figshare	233	3064	-	-	-	-
20	Badža and Barjaktarović [158] (2020)	2D	Figshare	233	3064 (Table 1)	Ax, Sag, Cor	T1c	Normalization, Resizing	Rotation, flipping
21	Banerjee et al. [56] (2020)	2D	TCGA-GBM TCGA-LGG BraTS2017	262 199 285 (Table 1)	1590 (LGG:750, HGG:840)	Ax ^1^ Ax, Sag, Cor ^2^	T1, T1c, T2, Flair	-	Rotation, shifts, flipping
22	Bhanothu et al. [179] (2020)	2D	Figshare	233	2406 (MN: 694, GL: 805, PT: 907)	-	T1c	Min-max normalization	-
23	Çinar and Yildirim [180] (2020)	2D	Kaggle-III	-	253 (Table 1)	Ax, Sag, Cor	-	-	-
24	Ge et al. [93] (2020)	2D	BraTS2017	285 (Table 1)	-	Ax, Sag, Cor	T1, T1c, T2, Flair	-	GAN
25	Ghassemi et al. [85] (2020)	2D	Figshare	233	3064 (Table 1)	Ax, Sag, Cor	T1c	Normalization (−1,1)	Rotation, flipping
26	Ismael et al. [159] (2020)	2D	Figshare	233 (Table 1)	3064 (Table 1)	-	T1c	Resizing, cropping	Rotation, flipping, shifting, zooming, ZCA whitening, shearing, brightening
27	Khan et al. [181] (2020)	2D	Kaggle-III	-	253 (Table 1)	-	-	Brain cropping	Flipping, rotation, brightness
28	Ma and Jia [129] (2020)	3D	CPM-RadPath2019	329 (Table 1)	329	Ax, Sag, Cor	T1, T1c, T2, Flair	Z-score normalization	Cropping, rotation, zooming, translation, color changes
29	Mohammed and Al-Ani [182] (2020)	2D	Radiopaedia	60 (15 per class)	1258 (H: 286, MN: 380, E: 311, Med: 281)	Ax, Sag, Cor	-	Resizing, denoising	Rotation, scaling, reflection, translating, cropping
30	Mzoughi et al. [142] (2020)	3D	BraTS2018	284 (LGG: 75, HGG: 209)	285	Ax, Sag, Cor	T1c	Min-max normalization, CE, resizing	flipping
31	Naser and Deen [183] (2020)	2D	TCGA-LGG	108 (G.II: 50, G.III: 58)	815 (G.II: 400, G.III: 415)	-	T1, T1c, Flair	Cropping, normalization (−1,1), resizing, segmentation	Rotation, zooming, shifting, flipping
32	Noreen et al. [184] (2020)	2D	Figshare	233	3064 (Table 1)		T1c	Normalization	-
33	Pei et al. [143] (2020)	3D	CPM-RadPath2020	270 (Table 1)	270	Ax, Sag, Cor	T1, T1c, T2, Flair	Noise reduction, z-score normalization, tumor segmentation	Rotation, scaling
34	Rehman et al. [104] (2020)	2D	Figshare	233	3064 (Table 1)		T1c	CE	Rotation, flipping
35	Saxena et al. [185] (2020)	2D	Kaggle-III	-	253 (Table 1)	-	-	Brain cropping, resizing	(20) not specified
36	Sharif et al. [186] (2020)	2D	BraTS 2013 ^1^ BraTS2015 ^2^ BraTS2017 ^3^ BraTS2018 ^4^	30 (Table 1) 274 (Table 1) 285 (Table 1) 284 (Table 1)	-	-	T1, T1c, T2, Flair	CE, tumor segmentation	-
37	Tandel et al. [105] (2020)	2D	REMBRANDT	112 (Table 1)	2132 (H: 1041, T: 1091) 2156 (H: 1041, LGG: 484, HGG: 631) 2156 (H: 1041, AS: 557, OG: 219, GB: 339) 1115 (AS-II: 356, AS-III: 201, OG-II: 128, OG-III: 91, GB: 339) 2156 (H: 1041, AS-II: 356, AS-III: 201, OG-II: 128, OG-III: 91, GB: 339)	Ax, Sag, Cor	T2	Skull-stripping	Rotation, scaling
38	Toğaçar et al. [96] (2020)	2D	Kaggle-III	-	253 (Table 1)	-	-	-	Oversampling
39	Vimal Kurup et al. [187] (2020)	2D	Figshare	233	3064 (Table 1)	-	T1c	Resizing	Rotation, cropping
40	Zhuge et al. [58] (2020)	2D	BraTS2018 TCGA-LGG	284 (Table 1) 30	284 (Table 1) 30	Ax, Sag, Cor	T1c, T2, Flair	Inhomogeneity correction, z-score normalization, min-max normalization, tumor segmentation	(23) - AutoAugment [170]
		3D	BraTS2018 TCGA-LGG	284 (Table 1) 30	284 (Table 1) 30	Ax, Sag, Cor	T1c, T2, Flair		Rotation, scaling, flipping
41	Alaraimi et al. [78] (2021)	2D	Figshare	233	3064 (Table 1)	-	-	HE, z-score normalization	Rotation, cropping, flipping, scaling, translation
42	Ayadi et al. [86] (2021)	2D	Figshare ^1^ Radiopaedia ^2^ REMBRANDT ^3^	233 (Table 1) - 112 (AS-II: 30, AS-III: 17, OG-II: 14, OG-III: 7, GB: 44)	3064 (Table 1) 121 (MN G.I: 36, GL G.II: 32, GL G.III: 25, GB: 28) -	Ax, Sag, Cor - -	T1c - -	- - -	(17) - Rotation, flipping, gaussian blur, sharpen
43	Bashir-Gonbadi and Khotanlou [188] (2021)	2D	IXI BraTS2017 Figshare Private	582 (healthy) 285 - -	- - 3064 230	-	-	Skull-stripping, resizing	Flipping, mirroring, shifting, scaling, rotation
44	Chakrabarty et al. [144] (2021)	3D	BraTS 2018 BraTS2019 LGG-1p19q Private	43 LGG 335 (Table 1) 145 1234 (MET: 710, MN: 143, AN: 158, PA: 82, H: 141)	43 335 159 1234	Ax, Sag, Cor	T1c	Co-registration, resampling, skull-stripping, z-score normalization, resizing	-
45	Decuyper et al. [140] (2021)	3D	TCGA TCGA-1p19q BraTS2019 GUH dataset	285 (LGG: 121, HGG: 164) 141 202 110	285 141 202 110	Ax, Sag, Cor	T1, T1c, T2, Flair	Tumor segmentation	Rotation, Flipping, Intensity scaling, Elastic transform
46	Díaz-Pernas et al. [151] (2021)	2D	Figshare	233	3064 (Table 1)	Ax, Sag, Cor	T1c	Z-score normalization	(2) Elastic transforms
47	Gab Allah et al. [94] (2021)	2D	Figshare	233	3064 (Table 1)	Ax, Sag, Cor	T1c	Normalization (−1,1)	(12) PGGAN ^1^ (9) Rotation, mirroring, flipping ^2^
48	Gilanie et al. [152] (2021)	2D	Private	180 (AS-1: 50, AS-II: 40, AS-III: 40, AS-IV: 50)	30,240 (AS-1: 8400, AS-II: 6720, AS-III: 6720, AS-IV: 8400)	T1 & Flair: Ax, T2: Ax, Sag	T1, T2, Flair	BFC, normalization, tumor Segmentation	Rotation
49	Gu et al. [189] (2021)	2D	REMBRANDT ^1^ Figshare ^2^	130 -	110,020 3064 (Table 1)	- -	- T1c	- -	- -
50	Guan et al. [153] (2021)	2D	Figshare	233 (Table 1)	3064 (Table 1)	Ax, Sag, Cor	T1c	CE, tumor ROI, min-max normalization	(3) Rotation, flipping
51	Gull et al. [154] (2021)	2D	BraTS2018 ^1^ BraTS2019 ^2^ BraTS2020 ^3^	- - -	1425 (LGG: 375, HGG: 1050) 1675 (LGG: 380, HGG: 1295) 2470 (LGG: 645, HGG: 1435, unknown: 390)	- - -	T1, T1c, T2, Flair T1, T1c, T2, Flair T1, T1c, T2, Flair	Grayscaling, median filtering, skull-stripping	-
52	Gutta et al. [106] (2021)	2D	Private	237 (G.I: 17, G.II: 59, G.III: 46, G.IV: 115)	660 (G.I: 27, G.II: 144, G.III: 184, G.IV: 305)	-	T1, T1c, T2, Flair	Resampling, co-registration, skull-stripping, tumor segmentation	-
53	Hao et al. [79] (2021)	2D	BraTS2019	335 (Table 1)	6700 (20 random slices per patient)	Ax, Sag, Cor	T1c, T1, T2	-	-
54	Irmak [190] (2021)	2D	RIDER REMBRANDT TCGA-LGG Figshare	19 (G.IV) 130 199 233	(total) 2990 (H: 1350, T: 1640) 3950 (H: 850, MN: 700, GL: 950, PT: 700, MT: 750) 4570 (G.II: 1676, G.III: 1218, G.IV: 1676)	-	T1c, Flair T1c, Flair T1c, Flair T1c	-	-
55	Kader et al. [191] (2021)	2D	BraTS2012 BraTS2013 BraTS2014 BraTS2015	- - - -	1000 1000 800 700	- - - -	- - - -	Noise removal, tumor segmentation, resizing	-
56	Kader et al. [192] (2021)	2D	Private	-	17,600	-	T1, T2, Flair	-	Yes, not specified
57	Kakarla et al. [193] (2021)	2D	Figshare	233	3064 (Table 1)	-	T1c	Resizing, min-max normalization, CE	-
58	Kang et al. [133] (2021)	2D	Kaggle-III ^1^ Kaggle-II ^2^ Kaggle-I ^3^	- - -	253 (Table 1) 3264 (Table 1) 3000 (Table 1)	- - -	- - -	Brain cropping, resizing	Rotation, flipping
59	Khan et al. [87] (2021)	2D	BraTS2015	274	169,880	Ax, Sag, Cor	T1, T1c, T2, Flair	Z-score normalization, tumor segmentation	(20) Rotation, zooming, geometric transforms, sharpening, noise addition, CE
60	Kumar et al. [88] (2021)	2D	Figshare	233	3064 (Table 1)		T1c	-	Rotation
61	Masood et al. [194] (2021)	2D	Figshare Kaggle-III	233 -	3064 (Table 1) 253 (Table 1)	- -	T1c -	BFC, CE, tumor ROI	-
62	Noreen et al. [134] (2021)	2D	Figshare	233	3064 (Table 1)	-	-	Min-max normalization	-
63	Özcan et al. [155] (2021)	2D	Private	104 (G.II: 50, G.IV: 54)	518	Ax, Sag, Cor	Flair	Multiple-cropping, z-score normalization	(20) Rotation, zooming, shearing, flipping, elastic gaussian transforms
64	Pei et al. [97] (2021)	3D	CPM-RadPath2020	256 (Table 1)	256 (Table 1)	Ax, Sag, Cor	T1, T1c, T2, Flair	BFC, z-score normalization	(oversampling)
65	Sadad et al. [195] (2021)	2D	Figshare	233 (Table 1)	3064 (Table 1)	Ax, Sag, Cor	-	CE, tumor detection	Rotation, flipping
66	Tandel et al. [107] (2021)	2D	REMBRANDT	130 (H: 18, T: 112)	2156 (H: 1041, T: 1091) 557 (AS-II: 356, AS-III: 201) 219 (OG-II: 218, OG-III: 91) 1115 (LGG: 484, HGG: 631)	Ax, Sag, Cor	T2	-	Rotation, scaling
67	Toğaçar et al. [80] (2021)	2D	Figshare	233	3064 (Table 1)	-	T1c	-	Rotation, scrolling, brightening
68	Yamashiro et al. [145] (2021)	3D	BraTS2018	284 (Table 1)	285 (Table 1)	Ax, Sag, Cor	T1c	Tumor segmentation	Flipping, scaling, shifting
69	Yin et al. [130] (2021)	3D	CPM-RadPath2020	256 (Table 1)	256 (Table 1)	Ax, Sag, Cor	T1, T1c, T2, Flair	Tumor segmentation, resizing, z-score normalization	Brightness, CE, saturation, hue, flipping, rotation
70	Aamir et al. [156] (2022)	2D	Figshare	233 (Table 1)	3064 (Table 1)	Ax, Sag, Cor	T1c	CE, min-max normalization, tumor ROI	(2) Rotation, flipping
71	Ahmad et al. [89] (2022)	2D	Figshare	233	3064 (Table 1)	-	T1c	Resizing, normalization	CDA: Rotation, scaling GDA: VAE, GAN
72	Alanazi et al. [160] (2022)	2D	Kaggle-I Kaggle-II ^1^ Figshare ^2^	- - 233	3000 (H: 1500, T: 1500) 3264 (Table 1) 3064 (Table 1)	-	-	Noise removal, cropping, z-score normalization, resizing	-
73	Almalki et al. [121] (2022)	2D	Kaggle-II ^1^ Figshare ^2^	- 233	2870 (H: 395, MN: 822, GL: 826, PT: 827) 3064 (Table 1)	- -	- -	Brain cropping, denoising, resizing	- -
74	Amou et al. [81] (2022)	2D	Figshare	233	3064 (Table 1)	Ax, Sag, Cor	T1c	Min-max normalization, resizing	None
75	Aurna et al. [82] (2022)	2D	Figshare Kaggle-II Kaggle [196]	233 - -	3064 (Table 1) 3264 (Table 1) 4292 (H: 681, MN: 1318, GL: 1038, PT: 1255)	- - -	- - -	Resizing	Rotation, flipping, zooming, shifting, scaling
76	Chatterjee et al. [59] (2022)	2D-3D	BraTS2019 IXI	332 (LGG: 73, HGG: 259) 259	332 259	2D: Ax, 3D: Ax, Sag, Cor	T1c	Skull-stripping, normalization (0.5,99.5), resampling	Affine, flipping
77	Chitnis et al. [197] (2022)	2D	Kaggle-II	-	3264 (Table 1)	-	-	Resizing	Autoaugment
78	Coupet et al. [135] (2022)	2D-3D	BraTS2018 BraTS2020	284 (Table 1) 369 (Table 1)	50,812	Ax	T1, T1c, T2, Flair	Histogram & min-max normalization	Rotation, deformations, shearing, zooming, flipping
79	Dang et al. [98] (2022)	3D	BraTS2019	335 (Table 1)	335 (Table 1)	Ax, Sag, Cor	T1, T1c, T2, Flair	Segmentation, gamma correction, window setting optimization	(oversampling) Rotation
80	Danilov et al. [146] (2022)	3D 2D	Private	707 (G.I: 189, G.II: 133, G.III: 127, G.IV: 258)	707 17,730	Ax, Sag, Cor Ax, Sag, Cor	T1c T1c	Z-score normalization, resampling ImageNet standardization	- Rotation, scaling, mirroring
81	Ding et al. [57] (2022)	2D-3D	Private TCIA + Private	101 (LGG: 58, HGG: 43) 50 (LGG: 25, HGG: 25)	3 slices as channels	Ax, Sag, Cor	T1c	Tumor ROI, normalization, resizing	-
82	Ekong et al. [198] (2022)	2D	BraTS2015 IXI Figshare	- - -	(total) 4000 (H: 1000, MN: 10,000, GL: 1000, PT: 1000)	- - -	- T1, T2 T1c	Resizing, normalization, denoising, BFC, registration, tumor segmentation	Shifting, Rotation, Brightening, Image enlargement, Flipping
83	Gao et al. [112] (2022)	3D	Private	39,210	39,210	Ax, Sag, Cor	T1, T2, T1c	Z-score normalization, resampling	-
84	Gaur et al. [199] (2022)	2D	Kaggle-II	-	2870	-	-	Resizing	Gaussian Noise
85	Guo et al. [150] (2022)	3D	CPM-RadPath2020	221 (Table 1)	221	Ax, Sag, Cor	T1, T1c, T2, Flair	BFC, skull-stripping, co-registration, tumor segmentation	Rotation, resizing, scaling, gaussian noise, CE
86	Gupta et al. [95] (2022)	2D	Kaggle-II	-	3264 (Table 1)	-	-	CE	CycleGAN
87	Gurunathan and Krishnan [200] (2022)	2D	BraTS	-	260 (LGG: 156, HGG: 104)	Ax, Sag, Cor	T1, T2	Resizing, tumor segmentation	Rotation, shifts, reflection, flipping, scaling, shearing
88	Haq et al. [90] (2022)	2D	Figshare	233 (Table 1)	3064 (Table 1)	-	T1c	Resizing	(2) Zooming
89	Hsu et al. [131] (2022)	3D	BraTS2020 CPM-RadPath2020	369 (Table 1) 270 (Table 1)	369 270	Ax, Sag, Cor	T1, T1c, T2, Flair	Sampling patches, z-score normalization, tumor segmentation	Rotation, flipping, affine translation
90	Isunuri and Kakarla [201] (2022)	2D	Figshare	-	3064 (Table 1)	-	T1c	Resizing, Normalization	-
91	Jeong et al. [113] (2022)	2D	BraTS2017	285 (Table 1)	1445 (largest slice ± 8)	Ax, Sag, Cor	T1, T1c, T2, Flair	Resizing, z-score normalization	Rotation, flipping
92	Kazemi et al. [108] (2022)	2D	Figshare ^1^ TCIA ^2^	233 20	1500 (MN: 1000, GL: 800, PT: 600) 8798	- -	T1c T1c	Resizing	-
93	Khazaee et al. [202] (2022)	2D	BraTS2019	-	26,904 (LGG: 13,671, HGG: 13,233)	-	T1c, T2, Flair	-	Rotation, flipping
94	Kibriya et al. [122] (2022)	2D	Figshare	233 (Table 1)	3064 (Table 1)	-	-	Min-max normalization, resizing	(5) Rotation, flipping, mirroring, adding noise
95	Koli et al. [203] (2022)	2D	Kaggle-III Figshare	- -	253 (Table 1) 3064 (Table 1)	- -	- -	-	Rotation
96	Lakshmi and Rao [204] (2022)	2D	Figshare	-	3064	-	T1c	-	-
97	Maqsood et al. [114] (2022)	2D -	Figshare BraTS2018	233 284 (Table 1)	3064 (Table 1) -	- -	T1c -	CE, tumor segmentation, z-score normalization	-
98	Murthy et al. [205] (2022)	2D	Kaggle-III	-	253 (Table 1)	-	-	Median filtering, CE, tumor segmentation	-
99	Nayak et al. [206] (2022)	2D	Figshare	-	3260 (196 H, 3064 Table 1)	Ax, Sag, Cor	T1c	Noise removal, gaussian blurring, min-max normalization	(21) Rotation, Shifting, Zooming
100	Rajinikanth et al. [124] (2022)	2D	TCIA	-	2000 (GL = 1000, GB = 1000)	Ax	-	-	-
101	Rasool et al. [125] (2022)	2D	Figshare	233	3064 (Table 1)	Ax, Sag, Cor	T1c	-	Yes, not specified
102	Raza et al. [207] (2022)	2D	Figshare	233 (Table 1)	3064 (Table 1)	Ax, Sag, Cor	T1c	Resizing	-
103	Rizwan et al. [208] (2022)	2D	Figshare REMBRANDT	230 (MN: 81, GL: 90, PT: 59) 70 (G.II: 32, G.III: 18, G.IV: 20)	3061 (MN: 707, GL: 1425, PT: 929) 513 (G.II: 204, G.III: 128, G.IV: 181)	Ax, Sag, Cor -	T1c T1c	Noise, cropping, resizing	(5) Salt-noise, grayscaling
104	Samee et al. [209] (2022)	2D	Figshare	236 (MN: 83, GL: 90, PT: 63)	3075 (MN: 708, GL: 1427, PT: 940)	Ax, Sag, Cor	T1c	Grayscaling	(16) Rotation, zooming, brightening
105	Samee et al. [147] (2022)	3D	BraTS2015	65 (LGG: 14, HGG: 51)	1056 (LGG: 176, HGG: 880)	-	T1, T1c, T2, Flair	Resizing, denoising, CE, tumor segmentation	-
106	Sangeetha et al. [210] (2022)	3D	Private	45	45	Ax, Sag, Cor	T2	Min-max normalization	(14) Rotation, translation
107	Saravanan et al. [109] (2022)	2D	BRATS REMBRANDT	274 135	1200 -	- -	- -	Resizing	-
108	Sekhar et al. [126] (2022)	2D	Figshare	233 (Table 1)	3064 (Table 1)	Ax, Sag, Cor	T1c	Min-max normalization, resizing	Yes but not specified
109	Senan et al. [99] (2022)	2D	Kaggle-II	-	3060 (H: 396, MN: 937, GL: 826, PT: 901)	Ax, Sag, Cor	-	Denoising, min-max normalization, resizing, CE	(H: 11, MN: 5, GL:6, PT: 5) Rotation, cutting, zooming, patching, padding, brightening
110	Srinivas et al. [211] (2022)	2D	Kaggle	-	256 (Benign: 158, Malignant: 98)	-	-	Brain cropping, z-score normalization, resizing	Scaling, cropping, resizing, flipping, rotation, geometric transforms
111	Tandel et al. [75] (2022)	2D	Rembrandt	112 (LGG = 44, HGG = 68)	-	Ax	T1, T2, Flair	None ^1^, Skull-stripping ^2^, tumor ROI ^3^	Scaling, rotation
112	Tripathi and Bag [83] (2022)	2D	TCIA	322 (LGG:159, HGG: 163)	7392 (LGG: 5088, HGG: 2304)	-	T2	Skull-stripping, segmentation	Rotation, flipping, scaling, cropping, translation
113	Tripathi and Bag [141] (2022)	3D	BraTS2019 TCGA-GBM TCGA-LGG LGG-1p19qdeletion [212]	202 158 119 138	(total) 617 (LGG: 331, HGG: 286)	Ax, Sag, Cor	T1c, T2, Flair	Co-registration, skul-stripping, resampling, tumor segmentation	Flipping, shifting, rotation, cropping
114	Tummala et al. [136] (2022)	2D	Figshare	233	3064 (Table 1)	Ax, Sag, Cor	T1c	-	-
115	Vankdothu et al. [213] (2022)	2D	Kaggle-II	-	3264	-	-	Grayscaling, rotation, denoising, tumor ROI	-
116	Wang et al. [132] (2022)	3D	CPM-RadPath2020	270	270	Ax, Sag, Cor	T1, T1c, T2, Flair	Resizing, brain cropping	Rotation, flipping, scaling, jittering
117	Xiong et al. [115] (2022)	2D	Private	211 (AS: 54, OG: 67, GB: 90)	633	Ax, Sag, Cor	ADC, T1c, Flair	Resampling, skull-stripping, z-score normalization, min-max normalization	-
118	Xu et al. [118] (2022)	2D	BraTS2020	369 (Table 1)	369	Ax	T1c, T1, T2	BFC, skull-stripping, registration, z-score normalization ^1^ tumor ROI ^2^	-
119	Yazdan et al. [214] (2022)	2D	Kaggle-II	-	3264 (Table 1)	-	T1, T2, Flair	Denoising	None
120	Zahoor et al. [103] (2022)	2D	Kaggle ^1^ Figshare ^2^	-	1994 (H) 3064	- Ax, Sag, Cor	- -	Resizing	Rotation, sharing, scaling, reflection
121	AlTahhan et al. [127] (2023)	2D	Figshare Kaggle-II Kaggle-I	- - -	2880 (H: 396, MN: 825, GL: 829, PT: 830)	- - -	T1c	-	-
122	Al-Zoghby et al. [137] (2023)	2D	Figshare	233	3064 (Table 1)	Ax, Sag, Cor	T1c	Resizing	-
123	Anagun [215] (2023)	2D	Figshare	-	3064 (Table 1)	Ax, Sag, Cor	T1c	Brain cropping, HE, denoising	(9) Flipping, rotation, shifting, zooming
124	Anand et al. [91] (2023)	2D	TCGA-LGG	110	3929	-	Flair	-	Flipping
125	Apostolopoulos et al. [216] (2023)	2D	Kaggle [217] Kaggle [218]	-	26,249 (H: 2000, MN: 7866, GL: 8208, PT: 8175)	-	-	-	-
126	Asif et al. [138] (2023)	2D	Figshare	233 (Table 1)	3064 (Table 1)	-	-	Resizing, denoising	-
127	Athisayamani et al. [110] (2023)	2D	Figshare	-	-	-	-	Denoising, skull-stripping, brain segmentation	Rotation, flipping
128	Bairagi et al. [111] (2023)	2D	BraTS2013 BraTS2015 OPEN-I NLM	-	65 327 229	-	T1, T2, Flair	Resizing	(40) Resizing, cropping, rotation, reflection, shear, translation
129	Deepa et al. [84] (2023)	2D	BraTS2018 ^1^ Figshare ^2^	- -	- 3064 (Table 1)	-	- T1c	Min-max normalization, tumor segmentation	Flipping, translation, rotation, brightening, CE, gaussian noise
130	El-Wahab et al. [219] (2023)	2D	Figshare	233	3064 (Table 1)	Ax, Sag, Cor	T1c	-	-
131	Hossain et al. [116] (2023)	2D	Kaggle-II	-	3264	-	-	-	(4) Rescaling, shearing, zooming, flipping
132	Hussain et al. [148] (2023)	3D	BraTS2020	369 (Table 1)	369 (Table 1)	Ax, Sag, Cor	T1, T1c, T2, Flair, Segmentation	Denosing, tumor segmentation	-
133	Kibriya et al. [119] (2023)	2D	Kaggle-III ^1^ Kaggle-I ^2^	- -	253 (Table 1) 3000 (Table 1)	- -	- -	- -	- -
134	Krishnapriya and Karuna [92] (2023)	2D	Kaggle-III	-	253 (Table 1)	-	-	Brain cropping	(Oversampling) Rotation, shifting, rescaling, mirroring
135	Kumar et al. [128] (2023)	2D	ACRIN-DSC-MR-BRAIN	-	1731	-	T1	Resizing, grayscaling, CE, tumor segmentation	-
136	Mahmud et al. [220] (2023)	2D	Kaggle-II CPTAC-GB ACRIN-FMISO-BRAIN	- 189 45	3264 (Table 1) - -	-	-	Normalization, smoothing	Mirroring, rotation, shifting, zooming
137	Muezzinoglu et al. [221] (2023)	2D	Kaggle-II	-	3264 (Table 1)	-	-	Resizing, patch division	-
138	Özkaraca et al. [222] (2023)	2D	Kaggle [223] (combines Figshare, Kaggle-I, Kaggle-II)	-	total: 7021 (H: 2002, MN: 1627, GL: 1623, PT: 1769)	-	-	-	-
139	Özkaya and Şağıroğlu [224] (2023)	2D	BraTS2020	369	(undersampling slices HGG)	Ax	T1c, T2, Flair	Tumor segmentation, min-max normalization	-
140	Rasheed et al. [225] (2023)	2D	Figshare	233	3064 (Table 1)	Ax, Sag, Cor	T1c	Resizing, normalization	None
141	Rui et al. [149] (2023)	2D	Private	42 (G.II: 18, G.III: 10, G.IV: 14)	1176 (G.II: 504, G.III: 280, G.IV: 392)	Ax	T1c, T2, Flair	Brain cropping, normalization	-
142	Shirehjini et al. [123] (2023)	2D	Private	58 (G.I: 8, G.II: 16, G.III: 10, G.IV: 22)	1061 (T1c: 229, T1: 251, T2: 299, Flair: 282)	Ax, Sag, Cor	T1, T1c, T2, Flair	Resizing, min-max normalization	-
143	Srinivasan et al. [226] (2023)	2D	REMBRANDT	-	3100	-	-	Denoising, tumor segmentation	-
144	Tandel et al. [139] (2023)	2D	REMBRANDT	112 (LGG: 44, HGG: 68)	-	Ax, Sag, Cor	T1, T2, Flair	Resizing	Rotation, scaling
145	van der Voort et al. [117] (2023)	3D	Erasmus MC [227] Haaglanden Medical Center BraTS REMBRANDT CPTAC-GBM Ivy GAP Amsterdam UMC Brain-tumor-progression University Medical Center Utrecht TCGA-LGG TCGA-GBM	816 279 168 109 51 39 20 20 6 107 133	(total) 1412 (G.II: 277, G.III:173, G.IV: 962)	Ax, Sag, Cor	T1, T1c, T2, Flair	Registration, resampling, BFC, skull-stripping, brain cropping, z-score normalization	(2) Cropping, rotation, brightening, CE
146	Wu et al. [157] (2023)	2D	BraTS2019	326 (LGG:76, HGG: 250)	slices with tumor	-	T1, T1c, T2, Flair	Z-score normalization, center-cropping	Rotation, translation, clipping

AS: Astrocytoma, Ax: Axial, BFC: Bias Field Correction, CE: Contrast Enhancement, Cor: Coronal, DA: Data Augmentation, E: Ependymoma, Flair: Fluid Attenuated Inversion Recovery, GL: Glioma, GAN: Generative Adversarial Network, GB: Glioblastoma, GDA: Generative Data Augmentation, H: Healthy, HE: Histogram Equalization, HGG: High-Grade Glioma, LGG: Low-Grade Glioma, Med: Medulloblastoma, MN: Meningioma, MT: Metastasis, OG: Oligodendroglioma, PT: Pituitary, ROI: Region of Interest, Sag: Sagittal, T: Tumor, T1c: T1 post-contrast weighted, VAE: Variational Auto-Encoder. *Numerical superscripts* link datasets with models in Table A2 when different data sources yield individual results.

**Table A2:** Model Overview: Comprehensive summary of the DL architectures employed across the reviewed papers. The table outlines key information, including the brain tumor classification task, data partitioning, architecture, and the reported performance metrics.

No.	Reference	Classification Task	Data Split			Architecture	Acc%	AUC%	F1%	Class Performance %
			Method	Ratio	Level					
1	Ge et al. [100] (2018)	LGG vs. HGG	Three-way	60:20:20	Patient	[T1c] CNN [T2] CNN [Flair] CNN [Modality-ensemble] CNN	83.73 69.74 75.40 90.87	- - - -	- - - -	LGG = 82.54, HGG = 84.92 LGG = 59.52, HGG = 80.15 LGG = 76.19, HGG = 74.60 LGG = 90.48, HGG = 91.27
2	Ge et al. [73] (2018)	LGG vs. HGG	Three-way	60:20:20	Patient	Custom CNN ^1^ [whole image] ^2^ [tumor ROI]	84.21 89.47	- -	- -	- LGG = 90.48, HGG = 86.67
3	Pereira et al. [74] (2018)	LGG vs. HGG	Three-way	60:20:20	Patient	Custom CNN ROI: brain, Std.: image ROI: brain, Std.: brain ROI: tumor, Std.: image ROI: tumor, Std.: brain	89.50 89.50 87.70 92.98	88.57 89.13 88.41 98.41	86.45 86.43 85.08 90.96	LGG = 80.00, HGG = 92.90 LGG = 80.00, HGG = 92.86 LGG = 86.67, HGG = 88.10 LGG = 86.67, HGG = 95.24
4	Yang et al. [101] (2018)	LGG vs. HGG	5-fold CV, Test	80:20	Patient	TL GoogLeNet TL AlexNet GoogLeNet AlexNet	94.50 92.70 90.90 85.50	96.80 96.60 93.90 89.40	- - - -	- - - -
5	Abd-Ellah et al. [167] (2019)	H vs. LGG vs. HGG	Three-way	65:10:25	-	Parallel CNNs	97.44	-	-	(R) 97.00, (S) 98.00
6	Anaraki et al. [168] (2019)	H vs. G.II vs. G.III vs. G.IV G.II vs. G.III vs. G.IV MN vs. GL vs. PT	Hold-out	80:20	-	Custom CNN + GA	93.10 90.90 94.20	- - -	- - -	H = 99.80, G.II = 88.40, G.III = 86.80, G.IV = 97.40 - MN = 87.80, GL = 98.30, PT = 96.5
7	Deepak and Ameer [76] (2019)	MN vs. GL vs. PT	5-fold CV		Patient	TL GoogLeNet -KNN -SVM -SoftMax	98.00 97.80 92.30	- - -	- 97.00 -	- MN = 96.00, GL = 97.90, PT = 98.90 -
8	Hemanth et al. [169] (2019)	MT vs. MN vs. GL vs. AS	-	-	-	Custom CNN	96.40	-	-	MT = 94.00, MN = 93.00, GL = 93.00, AS = 89.00
9	Kutlu and Avcı [120] (2019)	Benign vs. Malignant	5-fold CV	70:30	-	TL AlexNet-DWT -LSTM -SVM -KNN	98.66 92.09 85.91	99.00 - -	- - -	B = 99.33, M = 98.66 B = 96.04, M = 92.08 B = 92.95, M = 85.91
10	Lo et al. [102] (2019)	G.II vs. G.III vs. G.IV	10-fold CV		-	TL AlexNet AlexNet	97.90 61.42	99.91 82.22	- -	G.II = 96.90, G.III = 96.80, G.IV = 99.10 -
11	Muneer et al. [171] (2019)	G.I vs. G.II vs. G.III vs. G.IV	Hold-out	70:30	-	TL VGG19 Wndchrm	94.64 92.86	- -	93.71 92.32	- -
12	Rajini [172] (2019)	H vs. G.II vs G.III vs. G.IV MN vs. GL vs. PT	Hold-out	80:20	-	Custom CNN	96.77 98.16	95.65 97.93	93.54 97.21	H = 99.80, G.II = 89.20, G.III = 85.27, G.IV = 98.00 MN = 93.69, GL = 99.15, PT = 99.13
13	Rahmathunneesa and Muneer [173] (2019)	G.I vs. G.II vs. G.III vs. G.IV	Hold-out	70:30	-	TL AlexNet TL GoogLeNet TL InceptionV3 TL ResNet50	92.98 85.96 86.84 96.05	- - - -	96.06 91.71 91.62 97.76	G.I = 96.67, G.II = 93.44, G.III = 92.31, G.IV = 89.09 G.I = 86.67, G.II = 98.36, G.III = 63.46, G.IV = 92.73 G.I = 76.67, G.II = 93.44, G.III = 90.38, G.IV = 87.27 G.I = 93.33, G.II = 91.80, G.III = 100.00, G.IV = 100.00
14	Sajjad et al. [174] (2019)	G.I vs. G.II vs. G.III vs. G.IV MN vs. GL vs. PT	Three-way	50:25:25	-	TL VGG-19 w/o DA w/ DA w/o DA w/ DA	87.38 90.67 - 94.58	- - - -	- - - -	G.I = 90.03, G.II = 89.91, G.III = 84.11, G.IV = 85.50 G.I = 95.54, G.II = 92.66, G.III = 87.77, G.IV = 86.71 MN = 90.22, GL = 93.12, PT = 89.08 MN = 94.05, GL = 96.14, PT = 93.21
15	Sultan et al. [175] (2019)	MN vs. GL vs. PT G.II vs. G.III vs. G.IV	Hold-out	68:32	-	Custom CNN	96.13 98.70	- -	- -	MN = 95.50, GL = 94.40, PT = 93.40 G.II = 100, G.III = 95.00, G.IV = 100.00
16	Swati et al. [77] (2019)	MN vs. GL vs. PT	5-fold CV		Patient	Block-wise TL VGG19 Block-wise TL VGG16 TL AlexNet	94.82 94.65 89.95	- - -	91.73 91.50 86.83	GL = 95.97, MN = 89.98, PT = 96.81 (R) 93.51, (S) 94.56 (R) 89.10, (S) 89.84
17	Toğaçar et al. [176] (2019)	H vs. T	Hold-out	70:30	-	Custom CNN GoogLeNet AlexNet VGG16	96.05 89.66 87.93 84.48	98.00 - - -	94.12 90.32 88.52 85.25	H = 96.00, T = 96.08 H = 84.85, T = 96.00 H = 84.38, T = 92.31 H = 81.25, T = 88.46
18	Amin et al. [177] (2020)	H vs. T	Hold-out	50:50	-	Custom CNN	^1^ 97.00 ^2^ 98.00 ^3^ 100.00 ^4^ 96.00 ^5^ 97.00	- - - - -	- - - - -	H = 97.00, T = 97.00 H = 99.00, T = 95.00 H = 100.00, T = 100.00 H = 98.00, T = 92.00 H = 99.00, T = 93.00
19	Afshar et al. [178] (2020)	MN vs. GL vs. PT	Hold-out	80:20		Custom CNN	92.45	98.00	-	MN = 75.35, GL = 96.85, PT = 98.90
20	Badža and Barjaktarović [158] (2020)	MN vs. GL vs. PT	10-fold CV	60:20:20	Patient Patient Image Image	Custom CNN w/o DA w/ DA w/o DA w/ DA	84.45 88.48 95.40 96.56	- - - -	81.86 86.97 94.93 96.11	MN = 62.70, GL = 90.20, PT = 91.30 MN = 71.60, GL = 92.80, PT = 95.00 MN = 89.80, GL = 96.20, PT = 98.40 MN = 90.20, GL = 98.00, PT = 99.20
21	Banerjee et al. [56] (2020)	LGG vs. HGG	Hold-out		Patient	^2^ VolumeNet ^1^ SliceNet ^1^ PatchNet ^1^ TL ResNet ^1^ TL VGGNet	94.74 85.96 82.45 72.30 68.07	- - - - -	- - - - -	LGG = 94.29, HGG = 96.00 LGG = 80.00, HGG = 88.10 LGG = 74.67, HGG = 85.24 LGG = 72.06, HGG = 71.43 LGG = 69.33, HGG = 67.62
22	Bhanothu et al. [179] (2020)	MN vs. GL vs. PT	Hold-out	80:20	-	F-RCNN + VGG16	-	-	-	(P) GL = 75.18, MN = 68.18, PT = 97.28
23	Çinar and Yildirim [180] (2020)	H vs. T	-	-	-	Custom CNN ResNet50 DenseNet201 AlexNet InceptionV3 GoogLeNet	97.01 92.54 91.04 89.55 88.07 71.64	- - - - -	96.90 93.33 92.30 90.05 81.81 66.03	H = 94.70, T = 100.00 H = 89.74, T = 96.40 H = 85.71, T = 100.00 H = 87.17, T = 92.85 H = 81.81, T = 100.00 H = 66.03, T = 92.85
24	Ge et al. [93] (2020)	LGG vs. HGG	Hold-out	60:20:20	Patient	Modality-ensemble Semi-supervised CNN w/o DA w/ DA	89.53 90.70	- -	- -	LGG = 78.26, HGG = 93.65 LGG = 84.35, HGG = 93.01
25	Ghassemi et al. [85] (2020)	MN vs. GL vs. PT	5-fold CV		Patient Patient Image	Custom CNN w/o pre-training w/ GAN pre-training w/ GAN pre-training	91.70 93.01 95.60	- - -	90.54 92.10 95.10	MN = 79.86, GL = 94.96, PT = 95.67 MN = 84.82, GL = 94.92, PT = 96.92 MN = 89.98, GL = 96.83, PT = 97.93
26	Ismael et al. [159] (2020)	MN vs. GL vs. PT	Hold-out	80:20	Patient Image	ResNet50	97.82 99.34	- -	97.00 99.00	MN = 93.00, GL = 99.00, PT = 99.00 MN = 98.00, GL = 99.00, PT = 100.00
27	Khan et al. [181] (2020)	H vs. T	Three-way	70:20:10	-	Custom CNN VGG16 ResNet50 InceptionV3	100.00 96.00 89.00 75.00	100.00 96.00 89.00 75.00	100.00 97.00 90.00 74.00	H = 100.00, T = 100.00 H = 92.85, T = 100.00 H = 85.71, T = 92.86 H = 76.92, T = 73.33
28	Ma and Jia [129] (2020)	AS vs. OG vs. GB	Three-way	70:10:20	Patient	[WSI] 2D ResNet50 [MRI] 3D DenseNet121 [WSI-MRI] Ensemble 2D-3D	83.33 71.10 88.90	- - -	91.40 82.90 94.30	- - -
29	Mohammed and Al-Ani [182] (2020)	H vs. EP vs. MN vs. MB	Three-way	70:10:20	-	Custom CNN	96.00	-	-	-
30	Mzoughi et al. [142] (2020)	LGG vs. HGG	-	-	Patient	Custom CNN	96.49	-	-	-
31	Naser and Deen [183] (2020)	G.II vs. G.III	5-fold CV		-	TL VGG16	95.00	97.00	-	G.II = 98.00, G.III = 93.00
32	Noreen et al. [184] (2020)	MN vs. GL vs. PT	Hold-out	80:20	-	InceptionV3 DenseNet201	99.34 99.51	99.00 100.00	- -	MN = 99.00, GL = 100.00, PT = 100.00 MN = 99.00, GL = 100.00, PT = 99.00
33	Pei et al. [143] (2020)	AS vs. OG vs. GB	Three-way	67:11:22	Patient	Custom CNN	63.90	-	-	-
34	Rehman et al. [104] (2020)	MN vs. GL vs. PT	Three-way	70:15:15	-	AlexNet GoogLeNet VGG16 TL AlexNet TL GoogLeNet TL VGG16	97.39 98.04 98.69 95.77 95.44 89.79	- - - - - -	- - - - - -	- - - - - -
35	Saxena et al. [185] (2020)	H vs. T	Three-way	70:20:10	-	TL ResNet50 TL VGG16 TL InceptionV3	95.00 90.00 55.00	95.00 90.00 55.00	95.20 90.90 68.90	- - -
36	Sharif et al. [186] (2020) *	LGG vs. HGG	10-fold CV, Test	70:30	-	Ensemble TL InceptionV3-DRLBP	^1^ 98.30 ^2^ 97.80 ^3^ 96.90 ^4^ 92.50	- - - -	- - - -	- - - -
37	Tandel et al. [105] (2020) *	H vs. T H vs. LGG vs. HGG H vs. AS vs. OG vs. GB AS-II vs. AS-III vs. OG-2 vs. OG-3 vs. GB H vs. AS-II vs. AS-III vs. OG-2 vs. OG-3 vs. GB	2-fold CV, 5-fold CV, 10-fold CV		-	TL AlexNet	100.00 95.97 96.65 87.14 93.74	- - - - -	100.00 94.80 94.78 86.89 91.97	100.00 94.85 94.17 84.40 91.51
38	Toğaçar et al. [96] (2020) *	H vs. T	Hold-out	70:30	-	Ensemble TL AlexNet-VGG16-RFE-SVM TL AlexNet TL VGG16	96.77 90.32 87.10	- - -	96.77 89.89 87.23	(R) 97.83, (S) 95.74 (R) 95.24, (S) 86.27 (R) 87.23, (S) 86.96
39	Vimal Kurup et al. [187] (2020)	MN vs. GL vs. PT	Hold-out	80:20	-	Custom CNN	92.60	96.33	93.33	GL = 96.00, MN = 94.00, PT = 94.00
40	Zhuge et al. [58] (2020)	LGG vs. HGG	5-fold CV, Test	60:20:20	Patient	TL 2D ResNet50 w/o DA w/ DA 3D ConvNet	89.10 96.30 97.10	- - -	- - -	(R) 86.40, (S) 91.70 (R) 93.50, (S) 97.20 (R) 94.70, (S) 96.80
41	Alaraimi et al. [78] (2021)	MN vs. GL vs. PT	Hold-out	80:20	-	TL VGG16 TL GoogLeNet TL AlexNet	100.00 98.50 94.40	98.60 98.10 97.60	- - -	- - -
42	Ayadi et al. [86] (2021)	^1^ MN vs. GL vs. PT ^2^ G.I vs. G.II vs. G.III vs. G.IV ^3^ H vs. T ^3^ H vs. LGG vs. HGG ^3^ H vs. AS vs. OG vs. GB ^3^ AS-II vs. AS-III vs. OG-II vs. OG-III vs. GB ^3^ H vs. AS-II vs. AS-III vs. OG-II vs. OG-III vs. GB ^2^ G.I vs. G.II vs. G.III vs. G.IV ^3^ H vs. T ^3^ H vs. LGG vs. HGG ^3^ H vs. AS vs. OG vs. GB ^3^ AS-II vs. AS-III vs. OG-II vs. OG-III vs. GB ^3^ H vs. AS-II vs. AS-III vs. OG-II vs. OG-III vs. GB	5-fold CV	70:30	-	Custom CNN [w/o DA] Custom CNN [w/ DA]	94.74 90.35 100.00 95.00 94.41 86.08 92.09 93.71 100.00 97.22 97.02 88.86 95.72	- - - - - - - - - - - - -	94.19 90.38 100.00 91.35 92.89 86.85 89.84 93.88 100.00 95.45 95.75 87.52 91.76	MN = 89.68, GL = 94.46, PT = 99.03 G.I = 88.23, G.II = 93.33, G.III = 84.00, G.IV = 96.00 H = 100.00, T = 100.00 H = 100.00, LGG = 100.00, HGG = 70.00 H = 99.00, AS = 96.36, OG = 92.00, GB = 80.00 AS-II = 85.71, AS-III = 90.00, OG-II = 86.66, OG-III = 80.00, GB = 85.71 H = 100.00, AS-II = 85.71, AS-III = 90.00, OG-II =86.66, OG-III = 80.00, GB = 82.85 G.I = 90.79, G.II = 95.66, G.III = 90.84, G.IV = 98.22 H = 100.00, T = 100.00 H = 100.00, LGG = 98.40, HGG = 86.00 H = 99.80, AS = 97.09, OG = 90.40, GB = 93.71 AS-II = 88.50, AS-III = 94.00, OG-II = 96.00, OG-III = 62.00, GB = 90.85 H = 100.00, AS-II = 93.14, AS-III = 88.00, OG-II = 98.66, OG-III = 76.00, GB = 94.85
43	Bashir-Gonbadi and Khotanlou [188] (2021)	MN vs. GL vs. PT H vs. LGG vs. HGG H vs. AS vs. MN vs. PT vs. LGG vs. HGG	Three-way	-	-	Auto-encoder CNN	98.50 99.10 99.30	- - -	98.6 99.2 99	MN = 97.90, GL = 99.00, PT = 98.60 H = 98.10, LGG = 99.00, HGG = 97.70 H = 100.00, AS = 100.00, MN = 100.00, PT = 100.00, LGG = 96.60, HGG = 97.80
44	Chakrabarty et al. [144] (2021)	LGG vs. HGG vs. MT vs. PT vs. AN vs. H vs. MN	5-fold CV, Test	80:20	Patient	Custom CNN	91.95	96.93	93.86	LGG = 81.50, HGG = 87.00, MT = 98.60, PA = 100.00, AN = 100.00, H = 89.70, MN = 93.30
45	Decuyper et al. [140] (2021)	LGG vs. HGG	Three-way	73:11:16	Patient	Custom CNN	90.00	93.98	-	LGG = 89.80, HGG = 90.16
46	Díaz-Pernas et al. [151] (2021)	MN vs. GL vs. PT	5-fold CV		Patient	Custom CNN	97.30	-	-	GL = 99.00, MN = 93.00, PT = 98.00
47	Gab Allah et al. [94] (2021) *	MN vs. GL vs. PT	Three-way	70:15:15	-	^1^ VGG19 ^2^ VGG19	98.54 96.59	- -	- -	GL = 100, MN = 90.20, PT = 96.92 -
48	Gilanie et al. [152] (2021)	AS-1 vs. AS-II vs. AS-III vs. AS-IV	Hold-out	50:25:25	Patient	Custom CNN	95.56	-	-	(Acc) G.I = 99.06, G.II = 94.01, G.III = 95.31, G.IV = 97.85
49	Gu et al. [189] (2021)	^1^ AS vs. OG vs. GB ^2^ MN vs. GL vs. PT	5-fold CV	70:30	-	Custom CNN	97.64 96.34	- -	94.18 94.69	AS = 96.86, OG = 91.27, GB = 93.09 MN = 88.75, GL = 94.87, PT = 98.37
50	Guan et al. [153] (2021)	MN vs. GL vs. PT	5-fold CV	70:30	Patient	EfficientNet	98.04	-	97.79	MN = 96.89, GL = 97.82, PT = 99.24
51	Gull et al. [154] (2021)	H vs. T	10-fold CV, Test	70:10:20	Patient	GoogLeNet	^1^ 96.49 ^2^ 97.31 ^3^ 98.79	- - -	97.27 97.92 99.12	H = 94.17, T = 97.80 H = 95.83, T = 98.14 H = 97.37, T = 99.42
52	Gutta et al. [106] (2021)	G.I vs. G.II vs. G.III vs. G.IV	Three-way	70:15:15	Patient	Modality-ensemble CNN GrB RF SVM	87.00 64.00 58.00 56.00	- - - -	- - - -	G.I = 100.00, G.II = 82.35, G.III = 76.92, G.IV = 92.50 G.I = 0.00, G.II = 23.53, G.III = 42.31, G.IV = 90.74 G.I = 0.00, G.II = 35.23, G.III = 7.69, G.IV = 92.50 G.I = 33.00, G.II = 70.00, G.III = 34.62, G.IV = 72.00
53	Hao et al. [79] (2021)*	LGG vs. HGG	Three-way	60:20:20	Patient	AlexNet TL AlexNet	- -	71.93 79.91	- -	- -
54	Irmak [190] (2021)	H vs. T H vs. MN vs. GL vs. PT vs. MT G.II vs. G.III vs. G.IV	5-fold CV, Test	60:20:20	-	Custom CNN	99.33 92.66 98.14	99.95 99.81 99.94	- - -	H = 100, T = 98.80 H = 92.10, MN = 94.20, GL = 94.40, PT = 88.00, MT = 90.00 G.II = 97.91, G.III = 100, G.IV = 97.01
55	Kader et al. [191] (2021)	H vs. T	-	-	-	DWAE model	99.30	-	96.55	H = 96.90, T = 95.60
56	Kader et al. [192] (2021) *	H vs. T	5-fold CV		-	Custom CNN GoogLeNet AlexNet VGG16	99.25 89.66 87.66 84.48	- - - -	95.23 90.32 88.52 85.25	(R) 95.89, (S) 93.75 (R) 84.85, (S) 96.00 (R) 84.38, (S) 92.31 (R) 81.25, (S) 8.48
57	Kakarla et al. [193] (2021)	MN vs. GL vs. PT	5-fold CV, Test	80:20	-	Custom CNN	97.42	-	-	-
58	Kang et al. [133] (2021) *	H vs. T H vs. T H vs. MN vs. GL vs. PT	Hold-out	80:20	-	Ensemble TL CNNs DenseNet169-InceptionV3-ResNeXt50-AdaBoost DenseNet121-ResNeXt-MnasNet DenseNet169-ShuffleNet-MnasNet	^1^ 92.16 ^2^ 98.83 ^3^ 91.58	- - -	- - -	- - -
59	Khan et al. [87] (2021)	LGG vs. HGG	-	-	-	VGG19 (w/o DA) VGG19 (w/ DA)	90.03 94.06	- -	- -	LGG = 91.05, HGG = 84.03 LGG = 96.05, HGG = 89.09
60	Kumar et al. [88] (2021)	MN vs. GL vs. PT	5-fold CV		-	TL ResNet50 (w/o DA) TL ResNet50 (w/ DA)	97.48 97.08	- -	97.20 97.20	97.20 97.20
61	Masood et al. [194] (2021)	MN vs. GL vs. PT H vs. T	Hold-out	70:30	-	DenseNet-41-based Mask-RCNN	98.34 97.90	- -	- -	(Acc) MN = 97.81, GL = 98.62, PT = 98.60 (Acc) H = 98.06, T = 97.74
62	Noreen et al. [134] (2021) *	MN vs. GL vs. PT	10-fold CV		-	TL InceptionV3 Ensemble InceptionV3-KNN-SVM-RF TL XceptionV3 Ensemble Xception-KNN-SVM-RF	93.31 94.34 91.63 93.79	- - - -	92.67 - 90.00 -	MN = 84.00, GL = 95.00, PT = 98.00 - MN = 78.00, GL = 94.00, PT = 100.00 -
63	Özcan et al. [155] (2021)	G.II vs. G.IV	5-fold CV, Test	80:20	Patient	Custom CNN AlexNet GoogLeNet SqueezeNet	97.10 92.30 93.30 89.40	98.90 97.00 98.70 97.50	97.00 92.22 93.30 89.30	G.II = 98.00, G.IV = 96.30 G.II = 94.00, G.IV = 90.70 G.II = 98.00, G.IV = 88.90 G.II = 92.00, G.IV = 87.00
64	Pei et al. [97] (2021)	AS vs. OG vs. GB	Hold-out	85:15	Patient	[WSI] 2D CNN [MRI] 3D CNN [WSI-MRI] Ensemble 2D-3D CNNs	77.00 69.80 80.00	- - -	88.60 77.10 88.60	- - -
65	Sadad et al. [195] (2021) *	MN vs. GL vs. PT	Hold-out	80:20	-	Custom CNN	99.60	99.00	-	-
66	Tandel et al. [107] (2021) *	H vs. T AS-II vs. AS-III OG-2 vs. OG-3 LGG vs. HGG	5-fold CV		Patient	Ensemble TL AlexNet, VGG16, ResNet18, GoogleNet, ResNet50	96.51 97.70 100.00 98.43	96.60 97.04 100.00 98.45	- - - -	(R) 96.76, (S) 96.43 (R) 94.63, (S) 99.44 (R) 100.00, (S) 100.00 (R) 98.33, (S) 98.57
67	Toğaçar et al. [80] (2021)	MN vs. GL vs. PT	Hold-out	80:20	-	Custom CNN	-	-	96.22	MN = 94.81, GL = 98.48, PT = 95.38
68	Yamashiro et al. [145] (2021)	LGG vs. HGG	Hold-out	85:15	Patient	Custom CNN	91.30	92.7	-	LGG = 69.20, HGG = 100.00
69	Yin et al. [130] (2021)	AS vs. OG vs. GB	Hold-out	86:14	Patient	[WSI] 2D DenseNet [MRI] 3D DenseNet [WSI-MRI] Ensemble 2D-3D	88.90 82.00 94.40	- - -	94.30 85.70 97.10	- - -
70	Aamir et al. [156] (2022)	MN vs. GL vs. PT	5-fold CV		Patient	Custom CNN	98.95	-	97.98	MN = 97.31, GL = 99.51, PT = 99.34
71	Ahmad et al. [89] (2022)	MN vs. GL vs. PT	Three-way	60:20:20	-	ResNet50 (w/o DA) ResNet50 (w/ CDA) ResNet50 (w/ GDA) ResNet50 (w/ CDA+GDA)	72.63 77.52 92.30 96.25	- - - -	71.07 76.06 91.77 96.97	MN = 73.94, GL = 76.92, PT = 65.05 MN = 76.76, GL = 82.87, PT = 69.89 MN = 92.25, GL = 96.15, PT = 86.56 MN = 96.47, GL = 96.50, PT = 95.70
72	Alanazi et al. [160] (2022)	H vs. T MN vs. GL vs. PT	Three-way	80:20, ^2^ Test	-	TL (on Kaggle-I) Custom CNN	95.75 96.90	- -	- 99.00	- MN = 92.00, GL = 98.70, PT = 98.20
73	Almalki et al. [121] (2022) *	H vs. MN vs. GL vs. PT MN vs. GL vs. PT	Hold-out	80:20, ^2^ Test	-	Custom CNN-SVM	98.00 97.16	- -	- -	H = 94.70, MN = 97.30, GL = 98.80, PT = 99.40 MN = 99.20, GL = 94.71, PT = 99.40
74	Amou et al. [81] (2022)	MN vs. GL vs. PT	Hold-out	90:10	-	Custom CNN VGG16 VGG19 DenseNet201 InceptionV3 ResNet50	98.70 97.08 96.43 94.81 92.86 89.29	- - - - - -	98.60 96.60 95.56 93.60 92.00 89.00	MN = 97.00, GL = 99.00, PT = 99.00 MN = 97.00, GL = 96.00, PT = 99.00 MN = 93.00, GL = 97.00, PT = 99.00 MN = 85.00, GL = 97.00, PT = 100.00 MN = 82.00, GL = 97.00, PT = 96.00 MN = 57.00, GL = 77.00, PT = 98.00
75	Aurna et al. [82] (2022)	H vs. MN vs. GL vs. PT	LOOCV (on dataset)		-	2-stage Ensemble EfficientNetB0-ResNet50- Custom CNN	98.96	98.90	99.00	H = 100.00, MN = 99.00, GL = 98.00, PT = 99.00
76	Chatterjee et al. [59] (2022)	H vs. LGG vs. HGG	3-fold CV, Test	70:30	Patient	(2+1)D ResNet TL (2+1)D ResNet 2D-3D Mixed ResNet TL 2D-3D Mixed ResNet 3D ResNet18 TL 3D ResNet18	- - - 96.98 - -	- - - - - -	90.35 92.37 86.07 93.45 90.95 89.25	H = 99.04, LGG = 91.43, HGG = 82.29 H = 99.88, LGG = 91.08, HGG = 87.05 H = 97.69, LGG = 88.60, HGG = 75.05 H = 99.51, LGG = 93.19, HGG = 88.37 H = 99.44, LGG = 92.06, HGG = 82.89 H = 99.97, LGG = 85.52, HGG = 83.53
77	Chitnis et al. [197] (2022) *	H vs. MN vs. GL vs. PT	Hold-out	88:12	-	Custom CNN DenseNet101 VGGNet16 ResNet50	90.60 86.80 88.33 85.79	95.60 92.84 94.31 94.34	91.48 87.84 89.60 86.96	(R) 91.50, (S) 97.99 (R) 86.14, (S) 96.07 (R) 88.15, (S) 98.61 (R) 85.17, (S) 95.77
78	Coupet et al. [135] (2022) *	H vs. T	Three-way	70:15:15	Patient	Modality-ensemble TL CNNs TL 3DUNet	86.38 82.96	- -	- -	- H = 69.81, T = 96.44
79	Dang et al. [98] (2022)	LGG vs. HGG	Three-way	60:20:20	-	VGG	97.44	-	-	-
80	Danilov et al. [146] (2022)	LGG vs. HGG G.I vs. G.II vs. G.III vs. G.IV LGG vs. HGG G.I vs. G.II vs. G.III vs. G.IV	Three-way	80:10:10	-	(3D) DenseNet (2D) TL ResNet200e	67.00 83.00 61.00 50.00	76.00 95.00 73.00 72.00	- 80.25 - 35.00	(R) 58.00, (S) 78.00 G.I = 100.00, G.II = 63.00, G.III = 100.00, G.IV = 85.00 (R) 44.00, (S) 81.00 G.I = 56.00, G.II = 45.00, G.III = 32.00, G.IV = 47.00
81	Ding et al. [57] (2022) *	LGG vs. HGG	Hold-out		Patient	Radiomics VGG16 Ensemble Radiomics-VGG16-RF	74.00 60.00 80.00	82.20 71.20 89.80	- - -	(R) 80.00, (S) 68.00 (R) 68.00, (S) 52.00 (R) 84.00, (S) 76.00
82	Ekong et al. [198] (2022)	H vs. MN vs. GL vs. PT	Three-way	80:10:10	-	Bayesian CNN MobileNet AlexNet VGG16 ResNet50	94.32 93.42 92.75 89.51 86.58	- - - - -	94.00 94.00 93.00 91.00 86.00	H = 97.50, MN = 92.50, GL = 85.50, PT = 100.00 94.00 93.00 91.00 87.00
83	Gao et al. [112] (2022) *	18 types of tumors *	Three-way	72:24:4	Patient	DenseNet	81.20	92.00	-	(R) 87.60, (S) 84.90
84	Gaur et al. [199] (2022)	MN vs. GL vs. PT	Three-way	80:10:10	-	Custom CNN	85.37	-	-	-
85	Guo et al. [150] (2022)	AS vs. OG vs. GB	3-fold CV		-	Radiomics Modality-fusion DenseNet201 Modality-ensemble DenseNet201	83.70 84.60 87.80	87.00 88.30 90.2	83.40 84.60 87.80	(R) 70.40, (S) 89.90 (R) 73.10, (S) 93.00 (R) 77.20, (S) 93.00
86	Gupta et al. [95] (2022)	H vs. T MN vs. GL vs. PT	Hold-out	88:12	-	InceptionResNetV2-RF	96.66 96.88	- -	97.00 96.00	H = 100.00, T = 93.00 MN = 100.00, GL = 100.00, PT = 85.00
87	Gurunathan and Krishnan [200] (2022)	LGG vs. HGG	Hold-out	75:25	-	Custom CNN AlexNet VGG19 GoogLeNet	99.40 98.14 97.97 95.69	- - - -	98.10 - - -	(R) 97.20, (S) 98.60 - - -
88	Haq et al. [90] (2022) *	MN vs. GL vs. PT	Hold-out	70:30	-	[w/o DA] TL ResNet50 TL VGG-16 TL InceptionV3 [w/ DA] TL ResNet50 TL VGG-16 TL InceptionV3	99.10 98.78 97.78 99.89 98.98 98.50	98.78 98.06 97.00 99.56 97.98 98.76	99.50 97.49 97.39 99.43 98.79 98.00	(R) 89.60, (S) 100.00 (R) 84.64, (S) 99.80 (R) 92.23, (S) 96.88 (R) 96.13, (S) 99.08 (R) 97.87, (S) 100.00 (R) 98.56, (S) 100.00
89	Hsu et al. [131] (2022)	AS vs. OG vs. GB	Three-way	67:11:22	Patient	[WSI] 2D ResNet50 [MRI] 3D ResUNet [WSI-MRI] ResNet50-ResUNet	77.70 69.80 80.00	- - -	88.60 77.10 88.60	- - -
90	Isunuri and Kakarla [201] (2022)	MN vs. GL vs. PT	5-fold CV		-	Custom CNN	97.52	-	97.26	97.19
91	Jeong et al. [113] (2022)	LGG vs. HGG	5-fold CV		-	Custom CNN	90.91	96.34	-	(R) 92.69, (S) 84.90
92	Kazemi et al. [108] (2022) *	^1^ MN vs. GL vs. PT ^2^ G.II vs. G.III vs. G.IV	Hold-out	75:25	-	SVM-KNN AlexNet VGGNet AlexNet-VGGNet SVM-KNN AlexNet VGGNet AlexNet-VGGNet	80.14 91.88 89.96 98.06 82.44 92.59 90.05 98.99	80.93 92.67 90.29 99.14 84.63 92.9 90.51 99.23	- - - - - - - -	- - - MN = 98.10, GL = 98.88, PT = 98.50 - - - MN = 98.02, GL = 95.90, PT = 98.95
93	Khazaee et al. [202] (2022)	LGG vs. HGG	Hold-out	80:20	-	TL EfficientNetB0	98.87	-	-	(R) 98.86, (S) 98.79
94	Kibriya et al. [122] (2022)	MN vs. GL vs. PT	-	-	-	Ensemble AlexNet-GoogLeNet-ResNet18-SVM	99.70	100.00	-	MN = 99.80, GL = 98.96, PT = 100.00
95	Koli et al. [203] (2022)	H vs. T MN vs. GL vs. PT	Three-way	70:15:15	-	TL ResNet50	90.00 96.00	- -	90.00 95.00	- MN = 90.00, GL = 98.00, PT = 97.00
96	Lakshmi and Rao [204] (2022)	H vs. MN vs. GL vs. PT	Hold-out	80:20	-	InceptionV3	89.00	-	-	-
97	Maqsood et al. [114] (2022)	MN vs. GL vs. PT LGG vs. HGG	5-fold CV		-	TL MobileNetV2-SVM	98.92 97.47	98.93 -	97.87 96.71	MN = 99.03, GL = 98.82, PT = 98.79 (R) 97.22, (S) 97.94
98	Murthy et al. [205] (2022) *	H vs. T	-	-	-	Custom CNN	95.26	-	97.52	(R) 97.12, (S) 50.00
99	Nayak et al. [206] (2022)	MN vs. GL vs. PT	Hold-out	80:20	-	TL EfficientNet TL ResNet50 TL MobileNet TL MobileNetV2	98.78 96.33 96.94 94.80	- - - -	98.75 96.50 97.00 95.00	H = 98.00, MN = 100.00, GL = 97.00, PT = 100.00 H = 98.00, MN = 98.00, GL = 90.00, PT = 100.00 H = 98.00, MN = 95.00, GL = 94.00, PT = 100.00 H = 96.00, MN = 99.00, GL = 95.00, PT = 90.00
100	Rajinikanth et al. [124] (2022)	LGG vs. HGG	5-fold CV	90:10	-	TL VGG16-SoftMax TL VGG16-DT TL VGG16-KNN TL VGG16-SVM	96.50 96.00 96.50 97.00	- - - -	96.55 96.00 96.52 97.00	(R) 97.03, (S) 95.96 (R) 96.97, (S) 95.05 (R) 97.00, (S) 96.00 (R) 97.00, (S) 97.00
101	Rasool et al. [125] (2022)	H vs. MN vs. GL vs. PT	Hold-out	80:20	-	TL GoogLeNet GoogLeNet-SVM	93.10 98.10	-	-	H = 95.20, MN = 85.10, GL = 97.00, PT = 100.00 H = 98.70, MN = 97.30, GL = 97.80, PT = 98.90
102	Raza et al. [207] (2022)	MN vs. GL vs. PT	Hold-out	70:30	-	Custom TL GoogLeNet TL AlexNet TL GoogLeNet TL ShuufleNet TL ResNet50 TL MobileNetV2 TL SqueezeNet TL Darknet53 TL ResNet101 TL ExceptionNet	99.67 97.80 98.26 98.37 98.60 99.00 97.91 99.13 98.91 98.69	- - - - - - - - - -	99.66 97.66 98.33 98.33 98.33 99.00 97.66 99.00 98.66 98.00	(R) 100.00 (R) 97.66 (R) 98.66 (R) 98.66 (R)98.66 (R) 99.00 (R) 98.00 (R) 99.33 (R) 99.00 (R) 98.33
103	Rizwan et al. [208] (2022)	MN vs. GL vs. PT G.II vs. G.III vs. G.IV	Train, Val+Test	65:35	-	Custom CNN	99.80 97.14	- -	- -	(Acc) MN = 98.92, GL = 96.72, PT = 97.81 (Acc) G.II = 99.00, G.III = 96.00, G.IV = 99.00
104	Samee et al. [209] (2022)	MN vs. GL vs. PT	Hold-out	70:30	-	TL hybrid GoogLeNet-AlexNet TL AlexNet TL VGG16 TL MobileNetV2 TL ResNet TL SqueezeNet	99.10 96.00 95.00 95.00 94.00 92.00	99.00 97.00 95.00 95.00 94.00 92.00	- - - - - -	MN = 99.00, GL = 99.00, PT = 99.00 MN = 96.00, GL = 96.00, PT = 96.00 MN = 95.00, GL = 95.00, PT = 95.00 MN = 95.00, GL = 95.00, PT = 95.00 MN = 94.00, GL = 94.00, PT = 94.00 MN = 92.00, GL = 92.00, PT = 92.00
105	Samee et al. [147] (2022)	LGG vs. HGG	10-fold CV, Test	70:15:15	Patient	Custom CNN	88.60	-	-	LGG = 80.00, HGG = 88.60
106	Sangeetha et al. [210] (2022)	H vs. T	LOOCV		Patient	TL (in Rembrandt) CNN	94.00	-	-	(R) 85.00, (S) 73.00
107	Saravanan et al. [109] (2022)	^1^ LGG vs. HGG vs. PIT ^2^ OLI vs. EP vs. CAM	10-fold CV		-	SVM-RBF GoogLeNet CDbLNL SVM-RBF GoogLeNet CDbLNL	85.80 94.60 97.21 84.80 91.60 97.21	- - - - - -	85.10 90.90 95.72 84.10 90.10 94.34	(R) 81.90 (R) 91.50 (R) 95.62 (R) 80.90 (R) 91.50 (R) 93.86
108	Sekhar et al. [126] (2022)	MN vs. GL vs. PT	5-fold CV		Patient	TL GoogLeNet-SoftMax TL GoogLeNet-SVM TL GoogLeNet-KNN	94.90 97.60 98.30	- - -	94.30 97.35 97.24	MN = 96.92, GL = 91.13, PT = 97.77 MN = 97.96, GL = 94.59, PT = 100.00 MN = 94.57, GL = 98.02, PT = 99.10
109	Senan et al. [99] (2022)	H vs. MN vs. GL vs. PT	Hold-out	80:20	-	AlexNet-SoftMax AlexNet-SVM ResNet18-SoftMax ResNet18-SVM	93.30 95.10 93.80 91.20	- - - -	- - - -	H = 91.10, MN = 89.80, GL = 93.30, PT = 97.80 H = 91.10, MN = 89.80, GL = 93.30, PT = 97.80 H = 94.90, MN = 93.60, GL = 93.90, PT = 97.80 H = 87.30, MN = 93.60, GL = 93.30, PT = 97.20 H = 92.40, MN = 86.10, GL = 91.50, PT = 95.60
110	Srinivas et al. [211] (2022)	Benign vs. Malignant	Three-way	-	-	TL VGG16 TL InceptionV3 TL ResNet50	86.05 64.00 74.00	- - -	- - -	B = 89.47, M = 87.09 B = 5.55, M = 100.00 B = 89.47, M = 64.52
111	Tandel et al. [75] (2022) *	LGG vs. HGG	5-fold CV		-	TL Ensemble AlexNet, VGGNet, ResNet18, GoogLeNet, ResNet50 [Whole image] [Skull-stripped brain] [Tumor ROI]	98.43 98.63 99.06	98.45 98.63 99.07	- - -	(R) 98.33, (S) 98.57 (R) 98.63. (S) 98.57 (R) 99.04, (S) 99.10
112	Tripathi and Bag [83] (2022) *	LGG vs. HGG	Hold-out	70:30 80:20 90:10 Average	- - - -	DST Fusion TL ResNets	95.64 95.78 96.19 95.87	- - - -	92.41 91.91 94.13 92.82	(R) 92.12, (S) 95.97 (R) 95.12, (S) 95.10 (R) 96.95, (S) 95.77 -
113	Tripathi and Bag [141] (2022)	LGG vs. HGG	10-fold CV		Patient	Attention-based CNN	95.86	-	94.84	(R) 94.82, (S) 96.81
114	Tummala et al. [136] (2022)	MN vs. GL vs. PT	Three-way	70:10:20	-	Ensemble ViT	98.70	-	-	(R) 97.78, (S) 99.42
115	Vankdothu et al. [213] (2022)	H vs. MN vs. GL vs. PIT	Hold-out	88:12	-	CNN RNN CNN-LSTM	89.39 90.02 92.00	- - -	- - -	(R) 98.30 (R) 98.00 (R) 98.50
116	Wang et al. [132] (2022)	AS vs. OG vs. GB	Three-way	70:10:20	Patient	[WSI] Ensemble EfficientNet-B2, EfficientNet-B3, SE-ResNext10 [MRI] 3D CNN [WSI-MRI] 2D-3D Ensemble	82.20 73.30 75.00	- - -	88.60 82.90 75.30	-
117	Xiong et al. [115] (2022) *	AS vs. OG vs. GB	Three-way	70:15:15	Patient	[MRI] TL ResNet34 [MRI-tabular] TL ResNet34	67.50 70.00	- -	- -	AST = 85.70, OLI = 40.00, GBM = 68.80 AST = 85.70, OLI = 30.00, GBM = 81.30
118	Xu et al. [118] (2022) *	LGG vs. HGG	Three-way	60:20:20	Patient	^1^ TL ResNet18 ^1^ TL ResNet18+radiomics ^2^ TL ResNet18 ^2^ TL ResNet18+radiomics	83.33 88.10 87.40 94.10	- - - -	- - - -	(R) 90.8 (R) 90.1 (R) 93.1 (R) 97.1
119	Yazdan et al. [214] (2022) *	H vs. MN vs. GL vs. PT	k-fold CV		-	TL AlexNet TL ResNet Multi-scale CNN 1 Multi-scale CNN 2 Multi-scale CNN 3	87.89 91.98 89.27 94.19 89.67	- - - - -	88.03 91.59 89.41 94.06 89.49	(R) 87.86, (S) 85.42 (R) 91.44, (S) 89.79 (R) 89.15, (S) 86.91 (R) 93.74, (S) 92.62 (R) 89.24, (S) 88.35
120	Zahoor et al. [103] (2022) *	^1^ H vs. T ^2^ MN vs. GL vs. PT	Hold-out	60:40 80:20	-	ResNet18-Softmax TL ResNet18-Softmax TL ResNet18-SVM Custom CNN-SVM Custom CNN-SVM	97.43 98.91 99.16 99.56 99.20	- - - 99.90 -	97.56 98.69 98.94 99.45 99.09	(R) 98.12 (R) 99.66 (R) 97.99 (R) 98.99 MN = 98.60, GL = 99.30, PT = 99.50
121	AlTahhan et al. [127] (2023)	H vs. MN vs. GL vs. PT	Three-way	70:30:-	-	TL GoogLeNet-SoftMax TL AlexNet-SoftMax TL AlexNet-SVM TL AlexNet-KNN	88.00 85.00 95.00 97.00	- - - -	88.46 86.27 93.62 97.96	H = 87.50, MN = 88.00, GL = 88.50, PT = 88.00 H = 84.00, MN = 84.60, GL = 88.00, PT = 83.30 H = 92.60, MN = 92.30, GL = 100.00, PT = 96.00 H = 96.20, MN = 96.00, GL = 100.00, PT = 96.00
122	Al-Zoghby et al. [137] (2023)	MN vs. GL vs. PT	Hold-out	80:20	-	Ensemble TL VGG-16 & Custom CNN	99.00	99.00	99.00	MN = 98.00, GL = 100.00, PT = 99.00
123	Anagun [215] (2023)	MN vs. GL vs. PT	Three-way	80:10:10	-	TL EfficientNetv2 TL ResNet18 TL ResNet200d TL InceptionV4	99.85 99.62 99.83 99.69	99.92 99.75 99.84 99.73	98.07 96.64 97.72 97.19	98.05 96.71 97.66 97.37
124	Anand et al. [91] (2023)	H vs. T	Three-way	76:14:10	-	TL EfficientNetB0 TL InceptionV3 TL ResNet50 TL VGG19 Custom CNN w/o DA Custom CNN w/ DA Ensemble TL VGG19 & Custom CNN	- - - 95.00 96.00 97.00 98.00	- - - - - - -	54.50 91.50 85.00 96.00 96.50 97.00 98.50	H = 44.00, T = 30.00 H = 90.00, T = 94.00 H = 82.00, T = 81.00 H = 98.00, T = 96.00 H = 95.00, T = 98.00 H = 98.00, T = 96.00 H = 98.50, T = 99.00
125	Apostolopoulos et al. [216] (2023) *	H vs. MN vs. GL vs. PT	H10-fold CV		-	Attention VGG19 VGG19 ResNet152 MobileNetV2 InceptionV3	93.53 91.08 86.00 86.89 87.13	95.3 - - - -	90.55 - - - -	H = 99.60, MN = 90.62, GL = 96.76, PT = 91.61 - - - -
126	Asif et al. [138] (2023)	MN vs. GL vs. PT	Hold-out	80:20	-	TL Xception TL VGG16 TL DenseNet201 TL ResNet152V2 TL InceptionResNetV2 Ensemble TL DenseNet201, ResNet152V2, InceptionResNetV2	91.83 93.54 97.22 95.58 95.75 98.69	- - 98.00 98.00 96.00 99.00	90.65 93.01 96.81 95.12 94.96 98.39	MN = 82.98, GL = 92.63, PT = 97.31 MN = 84.40, GL = 96.49, PT = 97.31 MN = 92.91, GL = 98.60, PT = 98.39 MN = 92.91, GL = 94.74, PT = 98.92 MN = 89.36, GL = 97.54, PT = 97.85 MN = 96.45, GL = 99.29, PT = 99.46
127	Athisayamani et al. [110] (2023)	MN vs. GL vs. PT	-	-	-	TL ResNet152 CNN SVM	98.85 97.00 94.00	98.00 - -	- - -	MN = 97.00, GL = 98.00, PT = 99.00 (R) 94.00 (R) 94.00
128	Bairagi et al. [111] (2023) *	H vs. T	10-fold CV, Test	80:20	-	SVM TL AlexNet TL VGG16 TL GoogLeNet	89.53 98.67 90.67 91.49	- - - -	- - - -	- - - -
129	Deepa et al. [84] (2023) *	H vs. T	Hold-out	90:10	-	Custom CJHBA Based DRN	^1^ 92.10 ^2^ 91.84	-	- -	(R) 93.13, (S) 92.84 (R) 91.55, (S) 91.86
130	El-Wahab et al. [219] (2023)	MN vs. GL vs. PT	5-fold CV, Test	80:20	-	TL VGG16 TL VGG19 TL InceptionV3 TL ResNet50 TL MobileNet BTCfCNN TL BTCfCNN (bt folds) TL BTC-fCNN	92.07 93.05 80.35 74.48 89.16 93.08 98.63 98.86	- - - - - - - -	- - - - - 92.21 98.46 98.77	- - - - - (R) 92.01, (S) 96.34 (R) 98.49, (S) 99.31 (R) 98.83, (S) 99.41
131	Hossain et al. [116] (2023)	H vs. MN vs. GL vs. PT	three-way	80:10:10	-	TL InceptionV3 TL VGG16 TL Xception TL ResNet50 TL VGG19 TL InceptionResNetV2 Ensemble TL VGG16, InceptionV3, Xception	95.72 95.11 94.50 93.88 94.19 93.58 96.94	- - - - - - -	69.00 69.00 69.00 72.00 64.00 70.00 76.00	H = 100.00, MN = 98.00, GL = 31.00, PT = 70.00 H = 100.00, MN = 99.00, GL = 22.00, PT = 80.00 H = 98.00, MN = 91.00, GL = 39.00, PT = 77.00 H = 100.00, MN = 97.00, GL = 28.00, PT = 72.00 H = 100.00, MN = 97.00, GL = 22.00, PT = 64.00 H = 98.00, MN = 99.00, GL = 33.00, PT = 68.00 H = 100, MN = 93.00, GL = 49.00, PT = 73.00
132	Hussain et al. [148] (2023)	LGG vs. HGG	Hold-out	-	Patient	3D CNN -T1 -T1c -T2 -Flair -Segmentation Ensemble	94.00 94.00 94.38 93.23 94.38 94.20	- - - - - -	95.77 95.77 95.65 95.77 95.77 95.75	- - - - - -
133	Kibriya et al. [119] (2023)	H vs. T	Hold-out	70:30	-	^1^ Radiomics-SVM ^1^ Radiomics-KNN ^1^ VGG16-SVM ^1^ VGG16-KNN ^1^ Radiomics+VGG16-SVM ^1^ Radiomics+VGG16-KNN ^2^ Radiomics-SVM ^2^ Radiomics-KNN ^2^ VGG16-SVM ^2^ VGG16-KNN ^2^ Radiomics+VGG16-SVM ^2^ Radiomics+VGG16-KNN	72.00 84.00 92.10 88.10 93.30 96.00 96.10 96.00 98.00 97.80 99.00 98.70	- - - - 99.00 99.00 - - - - 100.00 100.00	- - - - 93.50 94.50 - - - - 99.00 99.00	- - - - 93.00 95.50 - - - - 99.00 99.00
134	Krishnapriya and Karuna [92] (2023)	H vs. T	Hold-out	70:30	-	[w/o DA] TL VGG16 TL VGG19 TL ResNet 50 TL InceptionV3 [w/ DA] TL VGG 16 TL VGG19 TL ResNet50 TL InceptionV3	90.50 90.70 88.02 66.26 99.00 99.48 97.92 81.25	- - - - - - - -	- - - - 99.08 99.17 82.24 58.16	- - - - 98.18 98.76 87.27 63.25
135	Kumar et al. [128] (2023) *	Benign vs. Malignant	Hold-out	90:10	-	ResNet50-Softmax ResNet50-SVM TL ResNet50	86.57 91.24 96.80	- - -	- - 97.34	- - Benign = 95.21, Malignant = 97.56
136	Mahmud et al. [220] (2023)	H vs. M vs GL vs. PT	Three-way	80:10:10	-	Custom CNN ResNet50 VGG16 InceptionV3	93.30 81.10 71.60 80.00	98.43 94.2 89.6 89.14	- - - -	91.13 81.04 70.03 79.81
137	Muezzinoglu et al. [221] (2023)	H vs. MN vs. GL vs. PT	10-fold CV		-	PatchResNet	98.10		98.01	H = 98.40, MN = 98.51, GL = 95.68, PT = 100.00
138	Özkaraca et al. [222] (2023)	H vs. MN vs. GL vs. PT	10-fold CV, Test	80:20	-	CNN VGG16 DenseNet Custom CNN	- - - -	- - - -	92.00 85.75 84.75 96.5	H = 98.00, MN = 84.00, GL = 90.00, PT = 97.00 H = 96.00, MN = 67.00, GL = 89.00, PT = 94.00 H = 99.00, MN = 83.00, GL = 99.00, PT = 58.00 H = 98.00, MN = 91.00, GL = 97.00, R PT = 99.00
139	Özkaya and Şağıroğlu [224] (2023)	LGG vs. HGG	10-fold CV		-	TL MobileNetV2 TL DenseNet201 TL Xception 99.63 TL InceptionV3 TL EfficientNetV2S 99.24	99.85 99.66 99.70 99.63 99.41	99.92 99.77 99.64 99.74 99.25	99.85 99.67 - 99.64 -	- - -
140	Rasheed et al. [225] (2023)	MN vs. GL vs. PT	Hold-out	80:20	-	Custom CNN VGG16 VGG19 ResNet50 MobileNet InceptionV3	98.04 90.70 92.82 94.77 93.47 85.97	98.00 93.00 94.00 96.00 95.00 88.00	98.00 90.00 93.00 95.00 93.00 85.00	MN = 95.00, GL = 99.00, PT = 100.00 MN = 79.00, GL = 92.00, PT = 99.00 MN = 85.00, GL = 94.00, PT = 98.00 MN = 89.00, GL = 95.00, PT = 99.00 MN = 90.00, GL = 92.00, PT = 99.00 MN = 66.00, GL = 89.00, PT = 98.00
141	Rui et al. [149] (2023) *	LGG vs. HGG	5-fold CV, Test	-	Patient	Inception CNN [Flair] [T1c] Modality-ensemble	69.00 74.00 80.00	- - -	60.00 70.00 78.00	(R) 75.00, (S) 60.00 (R) 75.00, (S) 73.00 (R) 76.00, (S) 87.00
142	Shirehjini et al. [123] (2023) *	G.I vs. G.II vs. G.III vs. G.IV	Three-way	70:15:15	-	TL VGG16-Softmax TL VGG16-LR TL-SVM	96.93 98.15 99.38	- - 99.93	96.64 98.12 99.09	(R) 99.29 (R) 97.94 G.I: 96.00, G.II = 100.00, G.III = 100.00, G.IV = 100.00
143	Srinivasan et al. [226] (2023)	H vs. MN vs. GL vs. PT	Hold-out	80:20	-	Custom CNN UNet ResNet	98.17 92.61 96.23	- - -	- - -	(R) 98.79, (S)91.34 (R) 97.56 (S) 81.51 (R) 97.90, (S) 90.23
144	Tandel et al. [139] (2023) *	LGG vs. HGG	5-fold CV		-	Ensemble TL AlexNet, VGG16, ResNet18, GoogLeNet, ResNet50	[T1] 94.75 [T2] 97.98 [Flair] 98.88	94.92 97.99 98.88	- - -	(R) 94.29, (S) 95.56 (R) 97.60, (S) 98.37 (R) 98.95, (S) 98.80
145	van der Voort et al. [117] (2023)	G.II vs. G.III vs. G.IV LGG vs. HGG	Three-way	75:15:15	Patient	UNet	71.00 84.00	81.00 91.00	-	G.II = 75.00, G.III = 17.00, G.IV = 95.00 (R) 72.00, (S) 93.00
146	Wu et al. [157] (2023)	LGG vs. HGG	Three-way	54:13:33	Patient	Attention-based custom CNN VGG19 ResNet50 DenseNet201 InceptionV4	95.19 - - - -	98.40 95.80 94.10 95.70 97.00	93.34 - - - -	(R) 94.01, (S) 99.53 - - - -

AS: Astrocytoma, Acc: Accuracy, AUC: Area Under the Receiver Operating Characteristic Curve, CDA: Classic Data Augmentation, CDbLNL: Convolutional Neural Network Database Learning with Neighboring Network Limitation, CJHBA: Chronological Jaya Honey Badger Algorithm, CNN: Convolutional Neural Network, CV: Cross-Validation, DA: Data Augmentation, DRLBP: Dominant Rotated Local Binary Patterns, DRN: Deep Residual Network, DT: Decision Tree, DCGAN: Deep Convolutional Generative Adversarial Network, DWAE: Deep Wavelet Auto-Encoder, DWT: Discrete Wavelet Transform, ELM: Extreme Learning, Machine, EP: Ependymoma, GA: Genetic Algorithm, GAN: Generative Adversarial Network, GB: Glioblastoma, GDA: Generative Data Augmentation, GL: Glioma, GL: Glioma, HGG: High-grade Glioma, KNN: K-Nearest Neighbors, LGG: Low-grade Glioma, LOOCV: Leave-One-Out Cross-Validation, LR: Logistic Regression, LSTM: Long Short-Term Memory, MB: Medulloblastoma, MN: Meningioma, MT: Metastasis, OG: Oligodendroglioma, PT: Pituitary, (P): Precision, (R): Recall, RF: Random Forest, RFE: Recursive Feature Elimination, ROI: Region of Interest, (S): Specificity, Std: Standardization, SVM: Support Vector Machine, TL: Transfer Learning, WSI: Whole Slide Image. *Papers highlighted with an asterisk* (*) indicate that not all outcomes are reported. For comprehensive details, readers are referred to the original paper. *Numerical superscripts* link models with datasets in Table A1 when different data sources yield individual results.
